# Synthesis and degradation of FtsZ quantitatively predict the first cell division in starved bacteria

**DOI:** 10.15252/msb.20188623

**Published:** 2018-11-05

**Authors:** Karthik Sekar, Roberto Rusconi, John T Sauls, Tobias Fuhrer, Elad Noor, Jen Nguyen, Vicente I Fernandez, Marieke F Buffing, Michael Berney, Suckjoon Jun, Roman Stocker, Uwe Sauer

**Affiliations:** ^1^ Department of Biology Institute of Molecular Systems Biology ETH Zurich Zurich Switzerland; ^2^ Department of Civil, Environmental and Geomatic Engineering Institute of Environmental Engineering ETH Zurich Zurich Switzerland; ^3^ Department of Biomedical Sciences Humanitas University Milan Italy; ^4^ Department of Physics University of California at San Diego La Jolla CA USA; ^5^ Microbiology Graduate Program Massachusetts Institute of Technology Cambridge MA USA; ^6^ Life Science Zurich PhD Program on Systems Biology Zurich Switzerland; ^7^ Department of Microbiology and Immunology Albert Einstein College of Medicine Bronx NY USA; ^8^ Section of Molecular Biology Division of Biological Science University of California at San Diego La Jolla CA USA

**Keywords:** division, *Escherichia coli*, FtsZ, protein degradation, starvation, Metabolism, Microbiology, Virology & Host Pathogen Interaction, Quantitative Biology & Dynamical Systems

## Abstract

In natural environments, microbes are typically non‐dividing and gauge when nutrients permit division. Current models are phenomenological and specific to nutrient‐rich, exponentially growing cells, thus cannot predict the first division under limiting nutrient availability. To assess this regime, we supplied starving *Escherichia coli* with glucose pulses at increasing frequencies. Real‐time metabolomics and microfluidic single‐cell microscopy revealed unexpected, rapid protein, and nucleic acid synthesis already from minuscule glucose pulses in non‐dividing cells. Additionally, the lag time to first division shortened as pulsing frequency increased. We pinpointed division timing and dependence on nutrient frequency to the changing abundance of the division protein FtsZ. A dynamic, mechanistic model quantitatively relates lag time to FtsZ synthesis from nutrient pulses and FtsZ protease‐dependent degradation. Lag time changed in model‐congruent manners, when we experimentally modulated the synthesis or degradation of FtsZ. Thus, limiting abundance of FtsZ can quantitatively predict timing of the first cell division.

## Introduction

The division of one cell into two daughters is a key feature of life, and we understand many molecular processes that achieve this fundamental biological event in different cell types. Less clear is the exact molecular basis to initiate the division process, especially in relation to nutrient input. Nutrition‐related cues proposed as decision signals include protein (Ward & Lutkenhaus, 1985) or DNA (Cooper & Helmstetter, 1968) concentrations, and metabolites that interact with the division machinery (Weart *et al*, 2007). Current models of bacterial division focus on exponential growth conditions (Willis & Huang, 2017) where nutrients are abundant. Typically, these models use phenomenological quantities such as biomass per cell as the decision input variable. For example, the adder model (Amir, 2014; Campos *et al*, 2014; Taheri‐Araghi *et al*, 2015; Soifer *et al*, 2016) accurately predicts that bacteria will divide after a constant amount of biomass addition after birth for exponential growth.

Before bacterial cultures can divide exponentially, individual cells must first make the decision for the initial division from a non‐dividing state, the typical situation for microbes in their natural environment (Wang & Levin, 2009). Moreover, in many environments, non‐dividing microbes receive nutrients only sporadically and in small quantities, such as in the gut (Koch, 1971), soil (Wang & Levin, 2009), ocean (Stocker, 2012), but often also in industrial fermentation processes (Löffler *et al*, 2016). The biomass per cell input variable is not sufficiently detailed to understand the decision process for the first cell division of a non‐dividing state. Furthermore, the biosynthetic capabilities of starved cells are generally not well understood (Liu *et al*, 2015). Hence, current models of cell division do not predict division timing for the widespread, naturally occurring sporadic nutrient conditions. Thus, open questions remain: What determines the onset of division following recovery from starvation? Which molecular entities affect their decision?

Here, we studied the first division decision of starved *E. coli* under sporadic nutrient supply. We developed methodologies to measure division occurrence and metabolic activity of starved cells under sporadic pulsing. We found that cells rapidly synthesized proteins and nucleic acids from pulsed glucose. By quantifying division timing as a function of sporadic glucose pulse frequency, we deduced that FtsZ abundance dynamics rate limits division, built a quantitative model, and substantiated it with follow up experiments.

## Results

### The lag time to division shortens with glucose pulse frequency for a subpopulation

**Figure 1:**
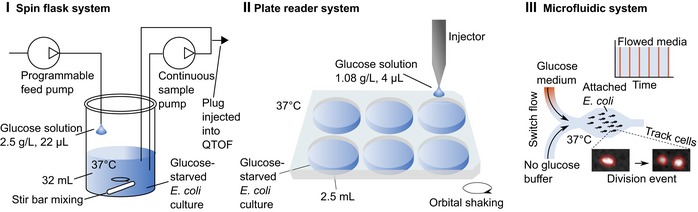
Schematics for nutrient pulse systems Three separate systems were used to pulse glucose to starved *Escherichia coli*. The spin flask system (I) and plate reader system (II) provided glucose pulses at defined frequencies. In the real‐time metabolomics configuration (Link *et al*, 2015), another pump circulated culture and injected 2 μl of culture directly into a time‐of‐flight (QTOF) mass spectrometer every 10–15 s from the spin flask system. A microfluidic platform (III) reproduced the pulse feeding and tracked division events. A pulsing period is defined as the time between the start of successive glucose medium exposures. During each pulse, glucose medium was flowed for 10 s, and the no glucose buffer was flowed in the intervening period.

We developed three complementary yet distinct systems (Fig 1) to controllably pulse nutrients to starved *E. coli* and measure division occurrence. Two of the systems (spin flask and plate reader) pulsed nutrients by dispensing a drop of defined volume at a programmed frequency to a starved culture. The drops were calibrated so that the final concentration, after the pulse mixed with the culture, was the same between the two systems. In the third system, bacteria attached to the bottom surface of a microfluidic chamber were suffused with flowed media and imaged over time. A pressure system controller allowed a precise and rapid switch of flowing medium and similarly provided nutrient pulses to the bacteria.

**Figure 2:**
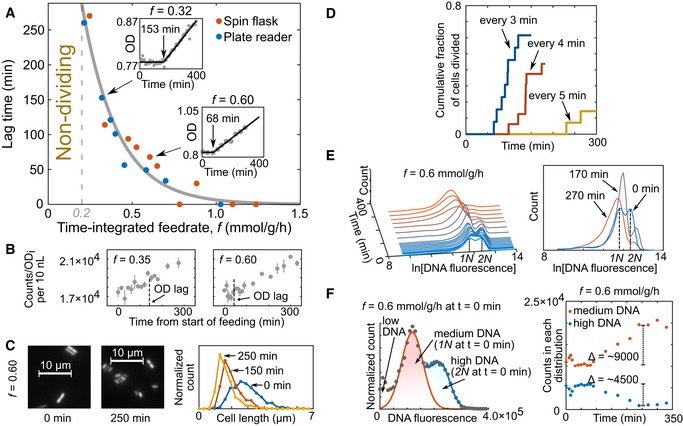
Lag time to division depends on the frequency of pulsed glucose for a subpopulation After 2 h starvation, *Escherichia coli* cultures were pulse‐fed 10 μM glucose at varying frequencies using the spin flask and plate reader systems, and optical density (OD) was measured over time (inset example figures). Gray dots are OD measurements, and the black lines are an empirical fit (see Materials and Methods). For separate experiments (*n* = 18), the lag time is plotted against the frequency, represented as the time‐integrated (TI) feedrate *f* (mmol glucose/g dry cell weight/h). An empirical fit (gray solid line, see Materials and Methods) was used to separate the lag (non‐dividing) and dividing phases. All OD data are summarized in Appendix Table S1.Normalized absolute cell counts versus time show linear increases after lag time for exemplary feedrates. Data are mean ± standard error of technical replicates (*n* = 2–3). Lag predicted from the empirical fit is indicated by vertical dotted lines.The average size of cells decreased after lag. Micrographs and cell length distributions (*n* > 400 per distribution) are shown for specific time points, with *f* = 0.6 mmol/g/h.Immobilized cells in the microfluidic experiment divided after a lag time that decreased with increasing glucose pulse frequency. The labeled times indicate the period, time between pulses, for a given experiment.Time course of the distribution of cellular DNA content. Sampled cells were stained with SYBR Green I and measured with flow cytometry over the course of a pulsing experiment (*f* = 0.6 mmol/g/h). Gating is shown in Appendix Fig S1. The DNA content distribution over time is shown on the left side, and three specific time points are shown on the right. Within the first time point (*t* = 0), the highest distribution is taken to be high DNA content (2N), and the distribution at half of the 2N average was taken (1N) as medium DNA.DNA distributions were separated into medium (1N) and high DNA (2N). Distribution‐specific estimated counts (see Materials and Methods) over time (*f* = 0.6 mmol/g/h) suggested that net division from high to medium DNA cells can explain the increase in cell counts and OD increase.

**Figure EV1:**
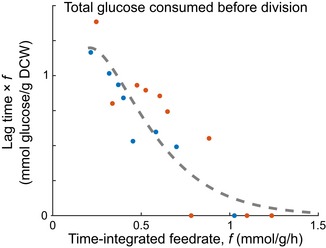
Total glucose fed during lag does not determine division occurrence Data from Fig 2A were replotted to total amount of glucose fed before division (lag duration times the TI feedrate) versus the TI feedrate. The total amount of glucose needed to trigger division is not constant and increases for decreasing TI feedrate.

How does sporadic nutrient availability empirically relate to the division decision? We focused on the case of limiting carbon and energy with sporadic glucose pulses. Glucose‐grown cells were starved for 2 h and then pulsed at controlled frequencies of 10 μM glucose with the spin flask and plate reader systems. Hereafter, we use the term time‐integrated (TI) feedrate (abbreviated *f*, units: mmol glucose/g dry cell weight/h) as the average rate of glucose fed over time normalized to the initial mass of cells in the culture. Our pulse frequency‐modulated TI feedrates spanned the range from 0.1 mmol/g/h, which does not support division, to just above 1 mmol/g/h. All TI feedrates were well below the exponential growth consumption rate of *E. coli* (~10 mmol/g/h; Monk *et al*, 2016). The cultures were glucose‐limited throughout the experiments, verified by absent glucose accumulation after pulses (Dataset EV1). To assess division occurrence as a function of TI feedrate, we measured the optical density (OD). Strikingly, the transition (lag) time to cell division, i.e., from constant to increasing OD, was dependent on pulsing frequency (Fig 2A and Appendix Table S1). The transition was not dictated by the total glucose fed, as the total glucose fed before division varied considerably across TI feedrates (Fig EV1). At TI feedrates below ~0.2 mmol/g/h (the critical rate), the OD did not increase within the first 6 h of pulsing. Above ~0.2 mmol/g/h, OD increase was only observed after a TI feedrate‐dependent lag time from the start of pulsing (Fig 2A insets). For TI feedrates above ~1.0 mmol/g/h, OD increased immediately without a detectable delay.

We confirmed that the OD increase reflects cell division by observing similar inflections in cell counts measured with flow cytometry occurring after lag times (Fig 2B). Since the total glucose fed during the lag phase was calculated to be insufficient for the doubling of the biomass of all cells in the culture (Appendix Table S1), we expected the average cell to become smaller. Indeed, microscopy demonstrated that the average cell size decreased after the onset of cell division (Fig 2C). Lastly, we used the microfluidic platform to similarly pulse feed cells and visually track division events (Fig 1 and Movie EV1). Consistent with our previous observations, division started after a lag time that shortened with increasing pulsing frequency (Fig 2D).

We noticed that not more than ~65% of the cells divided within 5 h in the microfluidic experiments, suggesting potential population heterogeneity. Furthermore, the initial linear increase in OD flattened before the initial OD was fully doubled (Appendix Fig S2). Both observations suggested that primarily a subpopulation undergoes the division. We hypothesized that this subset of cells was further along in the cell cycle before the start of pulsing compared to the rest. Therefore, we resolved the cell cycle status during pulsing by flow cytometric analysis of the DNA content distribution (Fig 2E). Before pulsing, two subpopulations existed, one with low (1N) and one with double DNA per cell (2N), as previously observed for *E. coli* in starved conditions (Akerlund *et al*, 1995). Upon glucose pulsing, the 2N cells disappeared while the 1N population increased. Members of the 2N population were in the D period (Wang & Levin, 2009) of the cell cycle before pulsing, with sufficient DNA for division yet limited nutritionally. Counting both 1N and 2N cells over time suggested that all division could be explained by 2N cells dividing into 1N (Fig 2F).

### Pulsed glucose is used rapidly to synthesize biomass even without division

**Figure 3:**
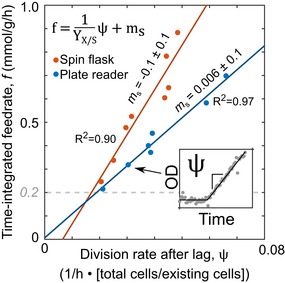
Maintenance metabolism alone cannot explain non‐division Linear decomposition of the TI feedrate (*f*) from data in Fig 2A separated the division (*Ψ*/*Y*_x/s_) and maintenance terms (*m*_s_): $f\;=\;\frac{1}{Y_{X/S}}\psi +m_{s}$. For the division term, the division rate (*Ψ*, units of 1/h · [number of new and existing cells]/[number of existing cells]) was calculated to be the slope after the lag ends (inset). For each pulsing system, the calculated yield (*Y*_x/s_, units of g DCW/mmol glucose · [number of new and existing cells]/[number of existing cells]) was constant and the extrapolated maintenance term (*m*_s_) was not significantly detected (*m*_s_ = −0.1 ± 0.1 mmol/g/h for spin flask system and *m*_s_ = 0.006 ± 0.1 mmol/g/h for the plate reader setup).

How is pulse‐fed carbon utilized during the lag phase? In principle, it could be consumed by non‐growth‐related maintenance requirements (Van Bodegom, 2007) or stored for division. We defined maintenance as any consumed glucose not used directly for division, but rather for energetic costs such as protein turnover and sustaining cell integrity. We wondered whether maintenance was equivalent to and explained the critical rate (~0.2 mmol/g/h), meaning that only fed glucose exceeding the maintenance could be utilized for division. We, therefore, decomposed the TI feedrate, *f*, into division and maintenance terms by assuming a linear dependence of the division rate (*Ψ*, units: 1/h · [number of new and existing cells/number of existing cells]) on the TI feedrate (Shuler & Kargi, 2001; Fig 3). The division rate was almost directly proportional to the TI feedrate, suggesting that the required maintenance (i.e., the y‐intercept) is less than the critical rate (~0.2 mmol/g/h) and generally too small for precise measurement, as seen before in carbon‐limited batch culture (Basan *et al*, 2015). We conclude that most carbon pulsed during lag is stored for eventual division and that the critical rate is not explained solely by maintenance requirement.

**Figure 4:**
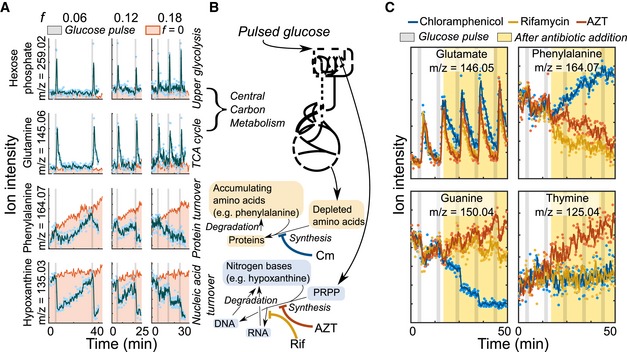
Glucose pulses induce brief, heightened protein and nucleic synthesis in non‐dividing *Escherichia coli* The spin flask system for glucose pulsing was connected to a real‐time metabolomics platform. Traces of exemplary ions are shown that correspond to hexose phosphate, guanine, phenylalanine, and hypoxanthine for pulsing at non‐division frequencies of 0.06, 0.12, and 0.18 mmol/g/h. The TI feedrate is abbreviated as *f* (units: mmol glucose/g dry cell weight/h). Glucose pulses are indicated by the gray bars, and the pink region shows a no pulse control. Dots are ion intensity measurements. Solid lines are a moving average filter of the measured ion intensity. For clarity, dots are not shown for *f* = 0 mmol/g/h condition.A metabolic scheme describing the propagation of fed glucose. Pulsed glucose is hypothesized to pass through central carbon metabolism and then be converted to downstream pathways including amino acid synthesis and nucleic acid synthesis. For nucleic acid synthesis, glucose is converted to the intermediate PRPP, which then can combine with nitrogen bases to form nucleotides for nucleic acid synthesis. Different pathways can be blocked with antibiotics. Color scheme used here accords to (C).Influence of antibiotics that inhibit macromolecular synthesis at the non‐division TI feedrate of 0.18 mmol/g/h. Antibiotics were added 1 min after the second pulse (yellow region). Four different ions are shown corresponding to glutamate, phenylalanine, guanine, and thymine. Chloramphenicol (blue) inhibits protein biosynthesis, rifamycin (orange) inhibits RNA polymerase, and azidothymidine (AZT; red) inhibits DNA synthesis. Ion traces with negative control (*f* = 0.18 mmol/g/h, no antibiotics) are shown in Appendix Fig S4.Data information: All ion data are available in Dataset EV1.

How do non‐dividing cells process and potentially store sporadically pulsed carbon? To address this question, we performed near real‐time metabolomics at a resolution of 10–15 s during the glucose pulses (Link *et al*, 2015). A continuous sample pump circulated culture liquid and provided 2 μl of whole cells in medium to a flow injector with time‐of‐flight mass spectrometer. More than 100 different annotated metabolites were measured (Dataset EV1). We observed sharply defined pulse responses in the concentration of all detected central metabolic intermediates (e.g., hexose phosphate and glutamine) at TI feedrates of 0.06, 0.12, and 0.18 mmol/g/h (Fig 4A and Appendix Fig S3A) that did not support cell division (Fig 2A). The concentration spike and the return to baseline levels within about 300 s strongly suggested a brief increase in central metabolic activity in response to pulsed glucose. Separately, several building blocks of cellular biomass such as amino acids and nitrogen bases continuously increased between pulses and rapidly decreased immediately after each glucose pulse (Fig 4A and Appendix Fig S3B). Since these accumulated amino acids included phenylalanine, which cannot be degraded by *E. coli*, their depletion suggested a brief increase in protein synthesis with each pulse (Caspi *et al*, 2016; Fig 4B). The nitrogen bases, hypoxanthine and guanine, may be salvaged for new nucleic acid synthesis upon sudden access to carbon. These observations suggested that fed carbon somehow ushers a brief, heightened biosynthesis of amino acid and nucleotide monomers and leads to a period of increased protein and nucleic acid synthesis immediately after the glucose pulses. This occurs even in the absence of cell division. Biosynthesis without division echoed earlier work about net protein synthesis in lag phase before division (Madar *et al*, 2013). Mechanistically, the glucose‐induced activity may be explained by a combination of phenomena: (i) The glucose pulse is directly conveyed into metabolism and sweeps through the network. The pulsed glucose moieties eventually form into the *de novo* biomass. (ii) Glucose stimulates increased metabolism through regulatory means (e.g., releasing the stringent response).

To confirm that protein and nucleic acid synthesis is engendered by fed carbon in non‐dividing cells, we repeated the glucose pulsing experiment but blocked macromolecule synthesis by adding antibiotics 1 min after the second pulse to curtail carbon to specific biosynthetic sectors (Fig 4C and Appendix Fig S3C). Upon addition of the ribosomal inhibitor chloramphenicol, the depletion of five measured amino acids including glutamate and phenylalanine was slowed compared to addition of other antibiotics. Conversely, guanine but not the amino acids exhibited a similar effect upon challenge with rifamycin and azidothymidine, which limit RNA and DNA synthesis, respectively (Cooper & Lovett, 2011). The DNA‐specific nitrogen base thymine, as expected, accumulated only upon azidothymidine addition.

**Figure 5:**
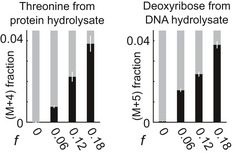
Glucose pulses incorporate directly into the *de novo* biomass in non‐dividing cells Percentage of labeled threonine and deoxyribose from protein and DNA hydrolysate shows *de novo* protein and DNA synthesis in non‐dividing cells. After 6 h of pulsing uniformly labeled ^13^C‐glucose, cultures were lysed, and their macromolecules were washed free of latent metabolites and hydrolyzed to monomers. Labeling data are presented as the mean ± standard error of independent biological replicates (*n* = 3, all pairwise *P* < 0.02 as determined by one‐sided Student's *t*‐test). All labeling data of all measured amino acids are available in Appendix Table S2.

To directly test whether pulsed glucose incorporates into biomass macromolecules at non‐division frequencies, we performed the same pulse experiments now with uniformly labeled ^13^C‐glucose instead. After feeding for 6 h, we harvested and lysed the cells. The soluble fraction was then washed multiple times with a cutoff filter to remove latent metabolites and to leave only macromolecules (e.g., protein and DNA). The macromolecules from the washed lysate were then hydrolyzed to monomer, which could be measured for labeled abundance. Increasing fractions of labeled threonine (M+4) and other amino acids in extracted and hydrolyzed protein confirmed *de novo* protein synthesis (Fig 5 and Appendix Table S2). Likewise, increasing labeled fractions of deoxyribose (M+5) from hydrolyzed DNA substantiated the use of pulsed carbon for *de novo* DNA synthesis (Fig 5) through the PRPP intermediate as shown previously (Link *et al*, 2015). Although glycogen is a storage form of glucose (Wilson *et al*, 2010), much less labeling was found in glycogen hydrolysate (Appendix Table S2). We conclude that pulsed glucose is not merely providing a regulatory effect—the pulsed glucose directly incorporates into the *de novo* biomass generated.

**Figure EV2:**
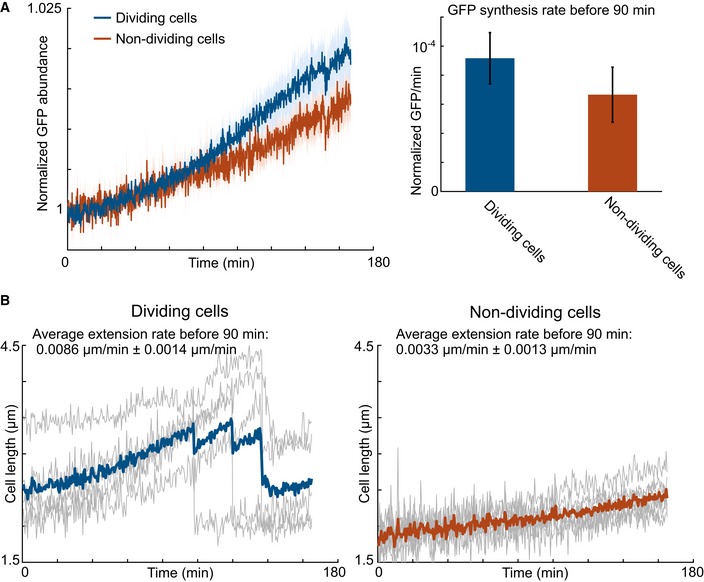
All cells make protein and increase in size with glucose feed Cell length and GFP expression were measured using the microfluidic‐based microscopy platform for glucose pulsing periods of 4 min. Two groups were determined: a dividing group (blue), where cells divided within the given experiment time (5 h), and a non‐dividing group (red), where the cells did not divide within the 5 h.
Both dividing and non‐dividing cells made protein as indicated by signal from constitutive GFP expression. Solid lines indicate the moving average for each given group. Lightly shaded regions indicate average ± standard error of the cells (*n* = 5). GFP synthesis rate was calculated before division (˜90 min) by using linear fitting. The bar graph shows the GFP synthesis rate for the both dividing and non‐dividing cells. Values are mean ± standard error of independent cells (*n* = 5).Cell length increased with pulsing in both the dividing versus non‐dividing subpopulation. Solid lines (blue and red) indicate the average of the individual cells. Gray lines indicate lengths of individual cells over time. Average cell extension rate before division was calculated using linear fitting for each cell. Values are mean ± standard error of independent cells (*n* = 5).

Lastly, we tested whether macromolecular synthesis occurred primarily in the 2N population using single‐cell microscopy under microfluidics with nutrient pulsing (Fig EV2). We separated populations of cells into dividing (all 2N) and non‐dividing. Dividing cells synthesized more biomass and protein before division compared to non‐dividing cells. Specifically, the cell elongation and GFP synthesis rates were higher in dividing cells (dividing cell extension rate of 0.0086 ± 0.0014 μm/min, dividing GFP synthesis rate of 9.2 × 10^−5^ ± 1.8 × 10^−5^ Norm. GFP/min versus non‐dividing cell extension rate of 0.0033 ± 0.0013 μm/min, non‐dividing GFP synthesis rate of 6.7 × 10^−5^ ± 1.9 × 10^−5^ Norm. GFP/min). Collectively, antibiotic challenges, ^13^C‐labeling, and microfluidics support our hypothesis that fed carbon is assimilated into protein and nucleic acids in non‐dividing cells during the lag phase.

### FtsZ synthesis and degradation limit division

**Figure 6:**
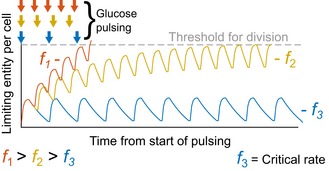
The limiting, degrading entity hypothesis The dependence of lag time on glucose pulse frequency can be explained with constitutive degradation of the limiting entity. In the model shown, the entity abundance is synthesized with each glucose pulse and depletes constitutively. Three example frequencies (*f*_1_ > *f*_2_ > *f*_3_) are shown where slight changes in period time dramatically change the time for the entity to reach the threshold needed to engender division. When synthesis and degradation of the entity are equal, the TI feedrate is at the critical rate (*f*_3_). Arrows indicate the glucose pulse frequency.

**Figure 7:**
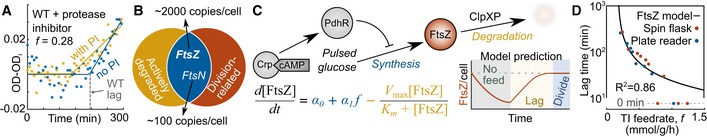
A dynamic model of FtsZ abundance predicts division timing Pulsing experiment was repeated in the presence of protease inhibitor (PI) that reduced the lag time for a given TI feedrate (*f* = 0.28 mmol/g/h). The TI feedrate is abbreviated as *f* (units: mmol glucose/g dry cell weight/h). Wild‐type lag (from Fig 2A empirical fit) is indicated by the dotted gray line.The sets of proteins that are actively degraded and division‐related intersect at FtsZ and FtsN.A schematic of how FtsZ abundance changes. FtsZ is repressed by the transcriptional factor, PdhR. PdhR is activated by Crp‐cAMP. FtsZ is also degraded primarily by the ClpXP protease complex. An approximate FtsZ threshold model poses a basal synthesis rate (*α*_*0*_), a feedrate‐dependent synthesis (*α*_*1*_ *f*), and a degradation term (the Michaelis Menten term) to explain changes in FtsZ abundance with and without pulsing. Per the model, FtsZ would deplete via degradation during starvation, be synthesized with glucose pulsing, and engender division when its abundance reaches the threshold concentration.Analytical solution of the model (Appendix Supplemental Information) plotted against data from Fig 2A (*R* ^2^ = 0.86). Lag time axis is log‐scaled.

Next, we asked what determines division occurrence. Since pulse‐fed glucose is converted into protein, RNA, and DNA in non‐dividing cells, we posit that, instead of an entire class of macromolecules, a specific molecule may stoichiometrically limit the division. Given that the lag time to the first division is a function of the pulse frequency, the most parsimonious explanation is that the limiting macromolecule is synthesized after the pulse for a brief period and constitutively degraded (Fig 6). This means that longer time between pulses results in more degradation and greater total glucose requirement to reach division, which is consistent with our data (Fig EV1). The competing synthesis and degradation can also explain the critical rate (~0.2 mmol/g/h); a critical rate would exist where the synthesis and degradation rates of the limiting entity are equal (*f*_3_ in Fig 6). Since proteins are the most abundant macromolecules (Milo *et al*, 2010) and because their degradation kinetics are consistent with the timescales observed (Sekar *et al*, 2016), we hypothesized that the limiting entity is a degraded protein. A key aspect of this theory is amenable to experimental validation: The lag time should be reduced by abrogating protein degradation with chemical protease inhibitors. We therefore added a cocktail of protease inhibitors at the onset of pulse feeding, using *f* = 0.28 mmol/g/h for which the usual lag time was about 200 min (Fig 7A). Consistent with our hypothesis of continuous degradation of one or more proteins that limit division, treatment with protease inhibitors reduced the lag time by 30%.

To identify the putative division limiting protein for division, we considered the known set of degraded proteins in *E. coli* (Flynn *et al*, 2003; Humbard *et al*, 2013), approximately 7% of the proteome. When we intersected the degrading protein set to the set of proteins involved in cell division (Zhou & Rudd, 2013), only FtsN and FtsZ remained (Fig 7B). Given that FtsN has very low abundance of around 100 copies per cell (Schmidt *et al*, 2016), we focused on FtsZ. FtsZ forms the division ring that septates a mother cell into two daughters (Adams & Errington, 2009). FtsZ is transcriptionally repressed by PdhR (Göhler *et al*, 2011), which is activated by the global transcriptional regulator Crp‐cAMP (Quail *et al*, 1994; Fig 7C). Since Crp‐cAMP regulation is highly active during carbon starvation in *E. coli* (You *et al*, 2013), one would expect *ftsZ* to be repressed during starvation and in the lag phase. Indeed, genetic disruption of *ftsZ* repression by deleting *crp* or *pdhR* entirely abrogated the non‐division phase, as cells divided without lag upon pulsing (Appendix Fig S5). These results suggest that FtsZ limits division and is synthesized during each pulse while being continuously degraded until its concentration reaches a level that supports division (Fig 7C inset). We tested the plausibility of this hypothesis by developing an approximate, smoothed dynamic model:

$$\frac{d[\text{FtsZ}]}{dt}\;=\;\alpha_{0}\;+\;\alpha_{1}f-\frac{V_{\max}\left[\text{FtsZ}\right]}{K_{m}\;+\;\left[\text{FtsZ}\right]}$$

The model accounts for the basal synthesis (*α*_0_), pulsing‐dependent synthesis (*α*_1_ *f*), and degradation (the Michaelis–Menten term) of FtsZ. We parameterized the model based on literature values mostly specific to FtsZ, the strain, and the media used (Camberg *et al*, 2009; Schmidt *et al*, 2016; Sekar *et al*, 2016; Appendix Supplemental Information). Despite fitting just a single parameter *α*_1_, the model reproduced non‐zero lag times remarkably well (*R* ^2^ = 0.86), supporting the role of FtsZ as the limiting entity for division (Fig 7D).

**Figure EV3:**
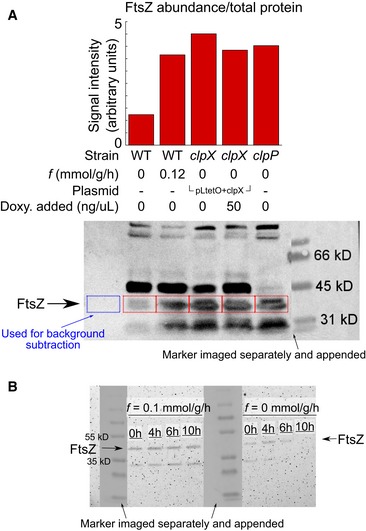
Western blots validate ClpXP‐mediated degradation of FtsZ *in vivo* during starvation and synthesis of FtsZ with glucose pulsing 1.5 ng total protein was loaded into each lane. Protein marker was imaged separately with bright‐field and appended to blot with exact positioning.
After 16 h, relative FtsZ abundance (from blot directly below) is much lower in wild‐type cells without any glucose pulsing (*f* = 0 mmol/g/h) compared to conditions with glucose pulsing (*f* = 0.12 mmol/g/h) or in strains absent of ClpXP machinery (*clpX* and *clpP*). Supplemental synthesis of ClpX within a *clpX* strain via expression off the pLtetO + clpX plasmid shows less FtsZ when ClpX synthesis is on (50 ng/μl doxycycline added) versus off (0 ng/μl doxycycline). Bordered areas were quantified with MATLAB 2015b. The subtracted background is indicated by the blue border.A time course immunoblot shows depletion of FtsZ in the no pulse condition (*f* = 0 mmol/g/h) versus the pulsing condition (*f* = 0.1 mmol/g/h) across 10 h. Time indicates sampling points from the beginning of pulsing (2 h into glucose starvation) for both experiments.

**Figure 8:**
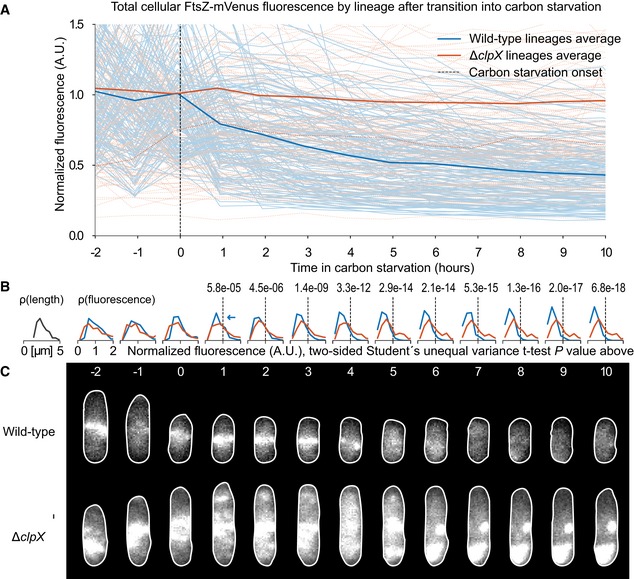
ClpX facilitates FtsZ depletion during carbon starvation Cells expressing a nearly functional FtsZ‐mVenus fluorescent fusion as their sole copy of *ftsZ* were monitored after transition into carbon starvation. Two low motile MG1655 strains, one (wild‐type) containing *clpX* and one without (*ΔclpX*), were grown in M9 glucose to steady‐state. At time zero, M9 media without glucose was flushed through device. Cells rapidly ceased elongation as measured by phase‐contrast imaging at 2‐min intervals. Fluorescent images were taken every hour throughout to measure both total Ftsz‐mVenus per cell and localization patterns. A time‐lapsed video of an experiment is available in Movie EV2.
Thin individual lines show the total fluorescent per cell in a single lineage (wild‐type in solid blue, *n* = 154. *ΔclpX* in dotted red, *n* = 144). Thick solid lines are the time average across individual lineages for each subset (wild‐type in blue, *ΔclpX* in red). After starvation, cells containing *clpX* degrade FtsZ faster than cells which do not. Fluorescent signals are normalized to the average value for their respective subset at time 0.Distributions of cellular fluorescence for each subset at times corresponding to the top panel. Cellular fluorescence varies widely as total FtsZ per cell is a function of cell size; a typical size distribution is shown at left. FtsZ concentration is roughly constant at steady‐state (Appendix Fig S6). Student's *t*‐test *P*‐value shown for when distributions differ with significance level α = 0.01.Representative images from cells in a single lineage with and without *clpX*. Wild‐type strains may contain the characteristic FtsZ ring at mid‐cell after shift down but it dissipates after several hours. *ΔclpX* strains display an abnormal FtsZ localization pattern even in steady‐state. After shift down, the FtsZ may disassemble and reform along the cell body in distinct puncta. Image timing corresponds to the abscissa in the top panel.

Our model postulates that FtsZ abundance depletes monotonically during starvation and increases upon glucose pulsing. Since resolving FtsZ abundance changes within a single pulse interval requires intractable sensitivity (FtsZ abundance changes ~1% between pulses, Appendix Supplemental Information), we monitored FtsZ abundance changes over longer periods with immunoblotting. Pulsing for 16 h at non‐division inducing TI feedrates yields several‐fold higher FtsZ concentrations compared to 16‐h starvation (Fig EV3), confirming that FtsZ is indeed one of the proteins synthesized from the glucose pulses under starvation. Deletion of the protease‐encoding genes *clpX* or *clpP* similarly increased FtsZ concentrations even under full starvation. Previous work has suggested that ClpX may not directly degrade FtsZ but rather inhibit FtsZ ring formation (Haeusser *et al*, 2009; Sugimoto *et al*, 2010). To explore this further, we performed mother machine experiments (Wang *et al*, 2010), where bacteria were entrained within microfluidic channels and imaged over time. The sole copy of *ftsZ* within the bacteria was fused to the fluorophore mVenus (Moore *et al*, 2017), yielding a nearly functional FtsZ‐mVenus. Fluorescence abundance and localization were measured during the transition into carbon starvation and many hours thereafter (Fig 8 and Movie EV2). We confirmed that the genetic presence of *clpX* facilitated the depletion of FtsZ in starvation, as shown in the wild‐type strain. In contrast, the depletion was nullified completely within the *clpX* mutant strain (Fig 8A). Given the variation in fluorescence, we confirmed that the distributions became statistically different between wild‐type and the *clpX* mutant after starvation (Fig 8B). We note that FtsZ localization appears distinct in the strain without ClpX (Fig 8C); specifically, fluorescent patterns indicate that FtsZ accumulates within puncta in the absence of ClpX, as opposed to localization at just the septum in the wild‐type strain. The abnormal localization supports the assertion that indeed ClpX may affect ring formation. Nonetheless, the immunoblotting, lag time dependence on pulse frequency, mother machine experiments, and protease inhibitor data all strongly indicate that FtsZ is degraded during starvation by ClpXP. This result is consistent with other evidence for ClpXP‐based degradation of FtsZ (Camberg *et al*, 2009; Pazos *et al*, 2013; Männik *et al*, 2018).

**Figure 9:**
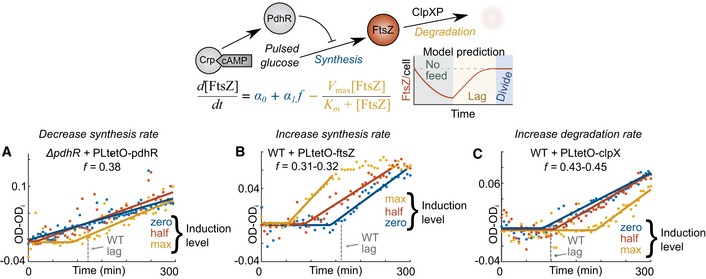
Experimental modulation of parameters supports the FtsZ limitation model Genetically induced titration of PdhR in a *pdhR* mutant reintroduced lag commensurate with expression level for a given TI feedrate (*f* = 0.38 mmol/g/h). Induction level corresponds to the amount of doxycycline (max – 50 ng/μl, half – 10 ng/μl, and zero 0 ng/μl) added at the onset of starvation. The TI feedrate is abbreviated as *f* (units: mmol glucose/g dry cell weight/h). Wild‐type lag (from Fig 2A empirical fit) is indicated by the dotted gray line.Lag time reduced with synthesis levels of titrated FtsZ in the wild‐type strain at *f* = 0.31–0.32 mmol/g/h.Lag time increased with titrated synthesis of ClpX in wild‐type cells at *f* = 0.43–0.45 mmol/g/h.

Observed synthesis and degradation of FtsZ alone, however, cannot establish its division limitation because many proteins are likely synthesized with glucose pulses and degraded. Instead, the model proffered clear, falsifying experiments to test FtsZ's candidacy as the limiting entity. We first titrated FtsZ synthesis, in effect modulating specific parameters while holding initial/division conditions, TI feedrate, and other parameters constant. At a TI feedrate of 0.38 mmol/g/h, a mutant strain with *pdhR* deletion that lacked FtsZ transcriptional repression divided without a lag phase, but the lag phase was gradually restored upon plasmid‐based expression of PdhR (decreasing *α*_0_ and *α*_1_; Fig 9A). Similarly, direct plasmid‐based supplementation of FtsZ (increasing *α*_0_ and *α*_1_) in the wild‐type reduced the lag time with increasing induction levels for a given TI feedrate (Fig 9B). The causal role of protein degradation was tested by modulating the FtsZ degradation rate through plasmid‐based overexpression of ClpX. ClpX abundance is known to be rate limiting for ClpXP‐based degradation (Farrell *et al*, 2005); therefore, supplemented ClpX should increase the FtsZ degradation rate (increasing *V*_max_). Consistent with our hypothesis, lag times prolonged at a given TI feedrate with increasing ClpX expression in *E. coli* (Fig 9C). We conclude that all titration experiments affecting the synthesis and degradation parameters are consistent with FtsZ division limitation.

**Figure EV4:**
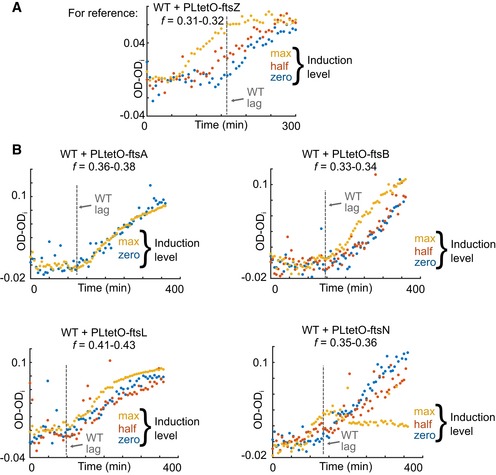
Titrations of other division proteins support FtsZ as the division limitation Figure 9B is reproduced here as reference and shows the decrease of lag time monotonic to the FtsZ induction level. All proteins were titrated via plasmid‐based, inducible expression. For induction, max, half, and zero correspond to addition of 50, 10, and 0 ng/μl of doxycycline, respectively. Units of TI feedrate *f* are mmol/g/h.Lag times do not decrease with induction level of other division proteins (FtsL, FtsB, and FtsA). FtsB and FtsL induction minimally increased more division after lag end. Lag time decreases with FtsN induction, and total division is decreased as shown by the lower final OD.

To exclude the possibility that also other division proteins are limiting, we titrated FtsA, FtsB, FtsL, and FtsN (Fig EV4). Overexpression of the former three did not affect the lag time, but at the highest induction level, FtsB and FtsL increased the division rate once the lag time ended. FtsN overexpression exhibited a more complex phenotype. While the highest induction level appeared to reduce the lag time, it also had a deleterious effect resulting in only a small increase in OD and thus presumably division of only very few cells. Therefore, the role of FtsN in the lag time to division remains inconclusive. We note that FtsN may have interaction effects with FtsZ (Addinall *et al*, 1997), thus affect lag through the FtsZ limitation model. We conclude that the negative controls do not falsify the FtsZ limitation model, but other division proteins may influence division through their interaction with FtsZ (Weiss, 2004) or may potentially be limiting in a smaller fraction of cells where FtsZ is sufficiently abundant.

In our model, the critical rate (*f* = 0.2 mmol/g/h) depends on the balance of FtsZ synthesis and degradation; thus, this critical rate should decrease if FtsZ synthesis is increased. We therefore performed pulse experiments at a feedrate of 0.17‐0.18 mmol/g/h, just below the critical rate, and not enough to trigger division within 6 h. While the OD of the control strain slowly decreases, overexpression of FtsZ indeed induced inflections in the OD commensurate with expression level (Appendix Fig S7), suggesting cell division proceeded. We thus conclude that increasing the FtsZ synthesis rate will decrease the critical rate threshold for division occurrence.

So far our pulsing experiments were performed with cells harvested from the mid‐exponential growth phase that were then subjected to a sudden starvation of 2 h. To exclude that our conclusions were influenced by the somewhat unnatural sudden starvation, we allowed the culture to enter a more “natural” starvation stage by slowly depleting glucose in the medium through consumption and entering a carbon‐limited stationary phase. The glucose pulsing experiment was then repeated with these cells after starvation for 2–6 h. Akin to the above‐reported experiments, FtsZ supplementation similarly decreased the lag time for the natural starvation condition (Appendix Fig S8).

**Figure EV5:**
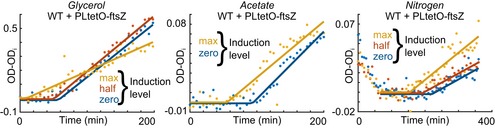
FtsZ‐limited division applies for various nutrient limitations Supplemental FtsZ titration reduced the lag time in starved cells that were pulse‐fed with the limiting nutrients glycerol, acetate, or ammonium. For induction, max, half, and zero correspond to addition of 50, 10, and 0 ng/μl of doxycycline, respectively. For the glycerol experiment, cells were grown in glycerol to exponential phase prior to starvation. Pulse concentration was 38 μM glycerol, and the TI feedrate was 0.6 mmol glycerol/g DCW/h where OD 1 corresponds to 0.54 g DCW/L (Gerosa *et al*, 2015). For the acetate experiment, cells were grown on acetate as the sole carbon source prior to starvation and pulse feeding. Pulse concentration was 88 μM sodium acetate at a TI feedrate of 3.0 mmol acetate/g DCW/h where OD 1 corresponds to 0.44 g DCW/L (Gerosa *et al*, 2015). For the ammonium experiment, cells were grown in M9 glucose media, then starved in media without ammonium, and consequently pulse‐fed. Pulse concentrations were 1.5 μM ammonium sulfate, and the TI feedrate was 0.045 mmol ammonium sulfate/g DCW/h.

Finally, we wondered whether FtsZ‐limited division is specific to pulsed glucose or a more general mechanism that links the nutritional status to the first cell division. For this purpose, we tested the influence of FtsZ overexpression on the lag phase upon pulsing carbon‐starved *E. coli* with the gluconeogenic carbon sources glycerol and acetate and nitrogen‐starved cells with the nitrogen source ammonium (Fig EV5). In all cases, FtsZ overexpression reduced the lag phase akin to the glucose case. Thus, our results suggest that the balance between FtsZ synthesis and protease‐mediated degradation is a general control mechanism for the first cell division during sporadic nutrient availability for a variety of different nutrients.

## Discussion

The rapid, unhindered biomass synthesis in non‐dividing, starved cells surprised us. Starved cells are expected to throttle metabolism and *de novo* biosynthesis (transcription, translation, and DNA replication) due to the stringent response (Liu *et al*, 2015) and, therefore, cease accumulating biomass (Jonas, 2014). Our expectation and the implicit one from earlier work anticipated that the metabolite pools must first replenish to continue the cell cycle and biosynthesis. Our measurements demonstrate that over a period of a few hours, glucose‐starved *E. coli* maintain a high anabolic and catabolic capacity. Furthermore, measured central carbon metabolite pools recrudesce and deplete within seconds, meaning even minuscule glucose passes through quickly. The limitation for division occurred on the protein level and not a specific metabolite, echoing recent work that argues the protein production and not metabolic activity limits cell cycle progression (Erickson *et al*, 2017; Towbin *et al*, 2017).

We demonstrated that, under conditions of sporadically available nutrients, the dynamics of FtsZ concentration primarily predicts the first cell division in *E. coli*. Finding limiting, key elements that engender division has been of long interest. The hypothesis that FtsZ concentration is limiting for division has been long hypothesized (Ward & Lutkenhaus, 1985), but later has only been applicable for downregulated FtsZ expression (Palacios *et al*, 1996) and cannot generally explain exponential growth conditions (Rueda *et al*, 2003; Weart & Levin, 2003) or otherwise. Such approaches did not account sufficiently for media conditions and nutrient consumption rate, which may have been relevant for potential FtsZ limitation. Separately, top‐down phenomenological, quantitative models have been generated (Willis & Huang, 2017), such as the recent adder model (Amir, 2014; Campos *et al*, 2014; Taheri‐Araghi *et al*, 2015) and the revived sizer model (Wallden *et al*, 2016), which predict division under a variety of media, species, and growth rates with few input variables. While such models offer parsimonious predictions in controlled environments, they lack molecular details. A quantitative model using molecular and nutrient information advances the field. Here, we generated a FtsZ‐based computational model that can quantitatively explain the timing of division for low levels of nutrient consumption (below ~1 mmol/g/h). This further suggests that the limitation of division in *E. coli* is consumption rate dependent and may be a key decision variable for other cell division models. In summary, we formulated the first quantitative model to predict bacterial division using molecular entity and nutrient input information.

## Materials and Methods

No statistical methods were used to predetermine sample size. The experiments were not randomized. The investigators were blinded to some sample measurements and outcome assessment.

### Strains and plasmids

*Escherichia coli* BW 25113 from the Keio collection (Baba *et al*, 2006) was used as the wild‐type (WT) strain for all experiments except the mother machine experiments. For mother machine experiments, strains contained *ftsZ*‐mVenus (Moore *et al*, 2017) in place of the endogenous *ftsZ* in a low motile MG1655 background. The *clpX* mutant was constructed via P1 transduction from the Keio knockout strain and confirmed via PCR. Kanamycin markers were excised from the Keio knockout strains *crp*,* pdhR* using pCP20 and verified using PCR (Datsenko & Wanner, 2000). All strains are listed in Appendix Table S3 and available from authors on request.

Plasmids are listed in Appendix Table S4, and all GenBank files are available in Dataset EV2. All plasmids originating from this study were designed using j5 software (Hillson *et al*, 2012), assembled using Gibson‐based techniques (Gibson *et al*, 2009), and sequence verified (Microsynth). Briefly, the titratable pJKR‐L‐tetR plasmid (Rogers *et al*, 2015) was used as a template where the sfGFP sequence was replaced with *pdhR*,* ftsZ*,* clpX, ftsA, ftsB, ftsL,* and *ftsN*. The plasmid pJKR‐L‐tetR was a gift from George Church (Addgene plasmid # 62561). For microfluidic experiments, the plasmid epd‐icd (Gerosa *et al*, 2013) was used for constitutive GFP expression. All plasmids originating from this study are available from AddGene (Article No. 25280).

### Cultivation, pulse feeding, and chemical concentrations

Cultivation procedure was followed as in an antecedent study (Link *et al*, 2015). All cultivation was performed at 37°C in shaker unless stated otherwise. Briefly, the day before pulsing, cells from freezer stock were cultivated in LB media for 3–5 h, then diluted 1:20 into 5 ml total of M9 media (see (Link *et al*, 2015) for recipe), and cultivated for 4–5 h to OD 0.1–0.2. 500 μl of the 5 ml inoculum was dispensed into 35 ml of M9 media and cultivated overnight at 30°C. The next day, cultures were typically at OD 0.2–0.3 and were then moved to 37°C and cultivated until OD 0.8–1.2. At this point, cells were pelleted by centrifugation (3 min at 5,000 *g*) and resuspended in 32 ml of diluted M9 media without glucose (1:8 dilution with filtered water). This point signified the start of starvation. Cultures were then cultivated without glucose for 2 h before the start of pulsing.

Glucose pulsing was accomplished using two systems (Fig 1). With the spin flask system, an IDEX Corporation Ismatec MCP 404 pump was programmed to dispense 22 μl of 2.5 g/l glucose solution to a 32 ml culture within a Schott bottle. Starved cultures were transferred to Schott bottle just before the start of pulsing. Frequency/flow rate was controlled by setting pause time between dispensations. Cultures were constantly mixed using a stir bar and maintained at 37°C by submergence into a water bath. Optical density (OD) was measured in a Pharmacia Novaspec II spectrophotometer. In the plate reader system, a Tecan Reader Infinite 200 with injector was programmed to dispense 4 μl of 1.08 g/l glucose solution to a 2.5 ml culture in 6‐well plates ((Thermo Fisher Scientific). Plate reader cultivations were performed at 37°C and with orbital shaking at maximum amplitude. An empirical function was used to convert OD measurements from the plate reader system to the spectrometer one.

Final concentrations of antibiotics were as follows: 100 μg/ml of ampicillin, 34 μg/ml of chloramphenicol, 50 μg/ml of rifamycin, and 100 ng/ml of azidothymidine. For plasmid titration experiments, doxycycline was added to the media at the onset of starvation. Each inducer concentration was cultivated in separate shake flasks during starvation. A titration curve is shown in Appendix Fig S9 for the plasmid expressing GFP. 50 ng/ml working concentration of doxycycline was used for maximal synthesis, 10 ng/ml for half synthesis, and none for zero synthesis. For the protease inhibitor experiment, a cOmplete™ EDTA‐free Protease Inhibitor Cocktail (Roche) tablet was dissolved in 2 ml of diluted media to form a stock solution. The stock solution was diluted 1:10 in M9 media without carbon source, inoculated with wild‐type *E. coli*, and cultivated for 2 days at 30°C to catabolize latent carbon within the cocktail solution. Cultivation was then pelleted, and the supernatant was collected and sterile filtered. Filtered, spent protease inhibitor solution was kept at 4°C for no more than 1 day before experiment. Spent protease inhibitor solution was warmed to room temperature and added 1:10 (total dilution of 1:100 from stock) at the onset of pulse feeding for the experimental condition. For the negative control condition, spent diluted M9 was added instead.

### Flow cytometry and DNA distribution analysis

Flow cytometry procedure was extended from a previous study (Berney *et al*, 2007). Two to three 5 μl samples were taken at every time point and diluted 1:10 in stain solution (filtered, spent media with 1:10,000 SYBR Green I and 1:5 propidium iodide). Stained samples were incubated for 10–15 min, diluted 1:100 in filtered, spent media (total dilution of 1:1,000), and then immediately measured in a BD Accuri C6 analyzer (BD Biosciences). 10 μl of diluted sample was injected at each time point, and the first three time points were used to calibrate the expected number of events (*E*_i_). Absolute counts for each sample (*C*_s_) were calculated by accounting for clogging in the sample injection port using the equation *C*_s_ = *E*_cells,s_/*E*_total,s_ · *E*_i_ where *E*_cells,s_ is the events in the gate (shown in Appendix Fig S1) for a given sample, and *E*_total,s_ is the total number of events in a sample. The instrument settings were the following: flow rate: slow; threshold limits: 800 on SSC‐H and 300 on FL1‐H. All data were exported to CSV tables and then gated and analyzed in MATLAB 2015b (MathWorks). FL1‐H was used for DNA fluorescence. DNA distribution peaks were separated by fitting a combination of two normal distributions in MATLAB 2015b.

### Fluorescence microscopy and image analysis

5 μl of samples was taken at every time point and diluted 1:10 in stain solution (filtered, spent media with 1:10,000 SYBR Green I). After 10 min, 12 μl of stained sample was deposited onto 2‐mm‐thick layer of 1% agar on top of a microscope slide. The agar with samples was dried under air flow, and a cover slip was placed and glued. The cells were then immediately imaged (phase and fluorescence) using a Nikon Eclipse Ti inverted epifluorescence microscope equipped with a CoolLED PrecisExcite light source and a Nikon 100× oil immersion objective. Filters used for fluorescence imaging of SYBR Green I were 505 nm (excitation) and 545 nm (emission). The exposure time was set to 12 ms. Cell lengths were calculated using the Straight and Segmented line tools in ImageJ. At least 400 cells were measured for each time point.

### Microfluidics and analysis

The WT strain with epd‐icd (Gerosa *et al*, 2013; constitutive GFP expression) was used for all microfluidic experiments. Non‐glucose buffer was diluted M9 media conditioned for 2 h with starved cells and then filtered. Glucose media was the non‐glucose buffer supplemented with 200 μM glucose. Cells were exposed to non‐glucose buffer for at least 2 h before glucose exposures to provide initial starvation. Microfluidic channels were 100 μm wide (where the cells were imaged) and 60 μm deep, with two inlet ports, a 5‐pointed junction, and two outlet ports. A pressure control system (Fluigent) allowed control of the duration and frequency of the glucose pulses. Before injecting the cells, the microfluidic devices were incubated with poly‐L‐lysine (Sigma, P8920; concentration 0.01% w/v) for 15 min to enhance the attachment of bacteria to the bottom glass surface of the channels (Rozhok *et al*, 2006). All experiments were performed using a Nikon Ti‐E inverted epifluorescence microscope equipped with Andor Zyla sCMOS camera, LED light sources (wavelengths 395, 440, 470, 508, 555, and 640 nm), a CAGE (LIS) incubator to maintain temperature at 37°C, and a Perfect Focus System to reduce focal drift during long acquisition times. Image analysis was performed in MATLAB (MathWorks) using in‐house cell tracking and identification algorithms. For calculations of GFP synthesis rate and cell extension rate, linear fitting was used on the data points for each cell before division.

### Mother machine experiments and analysis

Strains containing *ftsZ‐mVenus* either with or without *clpX* were plated on LB 2 days before the experiment and incubated at 37°C. Seed cultures in LB were begun the morning of the day before the experiment and back diluted into M9 glucose in the same day. Back dilutions were done such that cultures did not enter stationary phase. On the day of the experiment, cultures in exponential growth were concentrated and loaded in the mother machine device using a custom centrifuge. Device was passivated with 50 mg/ml bovine serum albumin before loading. After loading, the device was infused with fresh M9 glucose via a syringe pump (Harvard Apparatus, MA) and imaged at 37°C inside an environmental chamber (Darwin Chambers Company, MO). Imaging was performed on a Nikon Eclipse Ti inverted epifluorescence microscope (Nikon, Japan) with Perfect Focus 2, a 100× oil immersion phase objective (NA 1.45), an Andor Neo sCMOS camera (Andor Technology, UK), TLED diascopic light source (Sutter Instrument Company, CA), and 488LX Obis laser episcopic illumination (Coherent Inc., CA). Phase‐contrast images were taken every 2 min (30 ms exposure time). Fluorescent images were taken every hour (50 ms exposure time at 14 mW). Cells were grown in M9 glucose for at least 4 h before switching media to M9 without glucose via a second syringe pump and manual valve at the inlet to the device. Images were analyzed with custom software written in Python. Briefly, phase‐contrast images were used for cell segmentation and lineage reconstruction in order to calculate total fluorescence per cell.

### Real‐time metabolomics profiling, annotation of ions, and data normalization

Whole cell broth, real‐time metabolic profiling procedures were followed as in Link *et al* (2015). The ion annotation method is described in Fuhrer *et al* (2011). Ion suppression effects stemming from antibiotic addition are adjusted for as described in Link *et al* (2015). All ion intensity data were Z‐normalized and aligned (set *Z*) using the formula:

$$Z\;=\;\frac{S-\bar{S_{\mathrm{ref}}}}{\sigma_{\mathrm{ref}}}$$

*S* is the raw ion counts, $\bar{S_{\mathrm{ref}}}$ is the average of the reference set, and *σ*_ref_ is the standard deviation of the reference set. For comparison to the non‐pulsing condition (*f *= 0 mmol/g/h), the first 5 min were used for the reference set. For the antibiotic perturbations, the first 10 min were used for the reference set. All annotated ion data before Z‐normalization are available in Dataset EV1.

### Generation of washed lysates

Using the spin flask system, natively labeled cells were fed at TI feedrates of 0, 0.06, 0.12, or 0.18 mmol/g/h for 6 h with uniformly labeled ^13^C glucose. After the 6 h, 25 ml of the culture was sampled and pelleted, and supernatants were discarded. Pellets were stored at −80°C until extraction. Cells were lysed via resuspension into 2.5 ml of B‐PER solution (Thermo Fisher Scientific) and room temperature incubation for 10 min. 750 μl of lysates was clarified and spun through 10‐kDa size exclusion columns (Merck Millipore Ltd.). The flow through was discarded, and the retentate was resuspended in 200 μl of filtered ddH_2_O. In total, three such spin–wash steps were performed, and the final, washed retentate was used for further measurement.

### Protein hydrolysis and measurement

For protein hydrolysis and measurement, a previous protocol (Nanchen *et al*, 2007) was extended. The washed lysate was adjusted to 6 N by HCl addition. Acidified lysates were incubated for 1 h at 110°C and then dried under airflow at 65°C. Dried samples were silylated by dissolution in 50 μl dimethylformamide and then added to 100 μl L N‐tert‐butyldimethylsilyl‐N‐methyltrifluoroacetamide with 1% tertbutyldimethylchlorosilane. Reactions were then incubated at 85°C for 1 h. Products were measured on a 6890 GC combined with a 5973 Inert SL MS system (Agilent Technologies). Labeled fractions were adjusted for native isotope abundance (Nanchen *et al*, 2007).

### DNA hydrolysis and measurement

For measurement of deoxyribose derived from purified DNA, the PureLink Genomic DNA Mini (Thermo Fisher Scientific) kit was used to isolate DNA from the cleaned lysate. Of 0.5–1.0 μg of DNA was then hydrolyzed to nucleosides using the EpiQuik One‐Step DNA Hydrolysis Kit (Epigentek Group Inc). Reaction products were diluted to 100 μl with filtered water and spun through a size exclusion column. The flow through was directly measured on a 5500 QTRAP triple‐quadrupole mass spectrometer in positive mode with MRM scan type (AB Sciex). Nucleoside standards were used for compound optimization. The deoxyribose‐containing fragment of deoxyadenosine was measured for labeling fraction using SIM (*m*/*z* 252.3 > 117.2 through 257.3 > 122.2).

### Glycogen hydrolysis and measurement

For measuring glucose originating from glycogen, a previous method (Long *et al*, 2016) was extended. Washed lysate was acidified to 1 N by HCl addition in 300 μl total volume and incubated at 110°C for 1 h to hydrolyze polysaccharides. Samples were cooled on ice and neutralized with 84 μl of 3 N NaOH and then separated in size exclusion filters (10 kDa). The flow through was collected and dried in a SpeedVac setup (Christ) and precipitated with 500 μl cold ethanol. The ethanol resuspension was pelleted, and then, the supernatant was separated and dried overnight in the SpeedVac. Samples were dissolved in 50 μl of pyridine with 2% hydroxylamine hydrochloride and incubated for 1 h at 90°C. Samples were cooled to room temperature, and 100 μl of propionic anhydride was added. Mixed samples were incubated for 30 min at 60°C and then measured on the aforementioned GC‐MS system.

### Immunoblotting

For sampling, 500 μl of culture was collected and pelleted, and the supernatant was decanted. Samples were then immediately frozen at −20°C for no more than 1 week before blotting. On day of blotting, samples were resuspended in 50 μl B‐PER solution (Thermo Fisher Scientific) and incubated with shaking at room temperature for 10 min. Samples were pelleted, and protein concentration was determined by Bradford assay (Bio‐Rad) according to supplied protocol. 1.5 μg total protein was loaded into each well of a 4–12% polyacrylamide gel (Sigma), electrophoretically separated, and transferred to a nitrocellulose membrane (GE Healthcare). Membranes were blocked using TBS‐T buffer with 5% nonfat dry milk (Coop) for 1 h. Then, membranes were consequently incubated with prokaryotic Anti‐FtsZ primary antibody (Agrisera, Product No. AS10715) at 1:2,000 dilution in TBS‐T with milk overnight (4°C with agitation). The membrane was then washed three times with TBS‐T and incubated with HRP‐conjugated anti‐rabbit secondary antibody (Millipore, Product No. AP307P) at 1:10,000 dilution in TBS‐T with milk. Secondary incubation was conducted for 1 h, and then, the membrane was washed with TBS‐T three times. The membrane was then embrocated in Amersham ELC Prime Western Blotting Detection Reagent (GE Healthcare) to product specifications. After 5 min, the membrane was imaged first under bright‐field to visualize ladder lanes and then with chemiluminescence measurement for protein bands. All imaging was done with a gel imaging station (Bucher Biotec). Ladder lanes were appended to the image with protein bands using GIMP software with exact pixel alignment. Band quantification was performed with MATLAB R2015B (MathWorks).

### Calculations and fitting

MATLAB R2015B (MathWorks) or Python 2.7 was used for all calculations, fitting (using the *fitnlm* function in MATLAB), and data analysis. Optical density was converted to gram dry cell weight (DCW) with the conversion 1 OD = 0.4 g DCW/L, as determined for the strain and spectrophotometer specifically (Gerosa *et al*, 2015). For lag time (*t*_lag_), growth rate (*μ*), and initial cell amount (OD_i_) calculations, a threshold linear fit was applied to each OD versus time (*t*) plot:

$$\text{OD}\left(t\right)\;=\;\left\{\begin{array}{c} {\text{OD}}_{i}\;\text{for}\;t\;<\;t_{\mathrm{lag}}\\ \mu \left(t-t_{\mathrm{lag}}\right)\;+\;{\text{OD}}_{i}\;\text{for}\;t\geq \;t_{\mathrm{lag}} \end{array}\right.$$

OD values 3 standard deviations above the mean of each dataset were excluded from fits.

To empirically separate the non‐dividing and dividing phases (Fig 2A), a threshold exponential decay fit was used:

$$t_{\mathrm{lag}}\left(f\right)\;=\;\left\{\begin{array}{c} \text{indeterminate for}\;f\;<\;p_{0}\\ p_{1}\;\exp\;\left(-p_{2}\left(f-p_{0}\right)\right)\;\text{for}\;f\;\geq \;p_{0} \end{array}\right.$$

Correlation of coefficient (*R* ^2^) for the FtsZ model was calculated after log_10_ transformation of the lag times. Data that had 0 min lag time were excluded from the analysis.

## Data availability

All data and code used for figure generation are available in Dataset EV3 or at https://github.com/karsekar/pulsefeeding-analysis. In addition, the following dataset was deposited in a public resource:

- Flow cytometry: Zenodo https://doi.org/10.5281/zenodo.1035825.

## Author contributions

US and TF conceived the study. KS and US designed the experiments. US, RS, SJ, and MB supervised the work. KS developed and performed the spin flask, plate reader, flow cytometry, microscopy, real‐time metabolomics, and immunoblotting procedures. KS and JTS performed the molecular cloning procedures. KS, TF, and MFB developed and performed the labeling experiments. JN and VIF developed the microfluidic platform. RR performed the microfluidic experiments. JTS developed and performed the mother machine experiments. KS, RR, JTS, EN, and TF performed the calculations and analyzed the data. KS and EN developed the FtsZ model. KS, US, EN, JTS, and RR wrote the manuscript. All authors reviewed and approved the manuscript.

## Conflict of interest

The authors declare that they have no conflict of interest.

## Supporting information



Appendix

Expanded View Figures PDF

Dataset EV1

Dataset EV2

Dataset EV3

Movie EV1

Movie EV2

Review Process File
