# Supplementary material for: Synthesis and degradation of FtsZ quantitatively predict the first cell division in starved bacteria
Source: Mol Syst Biol. 2018 Nov 5;14(11):e8623. doi: 10.15252/msb.20188623 (PMC6217170; doi:10.15252/msb.20188623)
Supplement: Supplementary file 1 — Appendix [file MSB-14-e8623-s001.pdf]

Synthesis and degradation of FtsZ quantitatively predicts the first cell division in starved bacteria

Appendix

Karthik Sekar, Roberto Rusconi, John T. Sauls, Tobias Fuhrer, Elad Noor, Jen Nguyen, Vicente I. Fernandez, Marieke F. Buffing, Michael Berney, Suckjoon Jun, Roman Stocker, Uwe Sauer

Contents

1 Supplemental Figures 2

2 Supplemental Information - Development and parametrization of the FtsZ model 12
2.1 Introduction 12
2.2 Closed Form Solution for the Michaelis-Menten Equation 12
2.3 Applying the Closed Form Solution 13
2.4 Parametrization 14
2.5 Fitting α_1 15
2.6 Taking the pulsing into account 16

3 Supplemental Tables 17

References 20

1 Supplemental Figures

Appendix Figure S1: Gating for flow cytometry experiments.

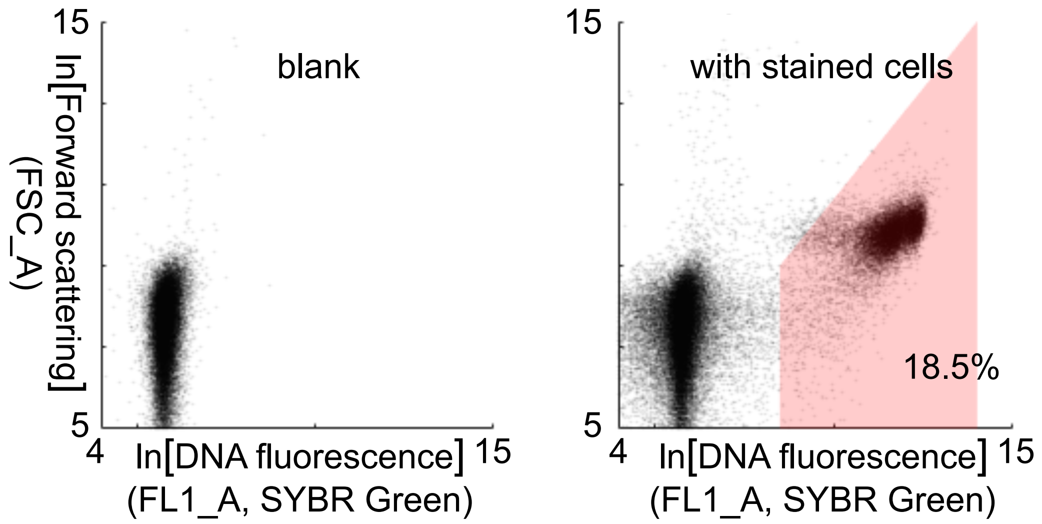

For all flow cytometry experiments, cells were stained with SYBR Green I dye and incubated for at least 10 minutes before measurement (see **Methods**). 10 µL of events were measured (scattering and fluorescence) at the slow rate (14 µL/min) and then analyzed with MATLAB R2015B. A scatter plot of the measured events are shown for a blank (left) and cell sample (right). We only focused on the forward scattering and green fluorescence dimensions. The gating used for all samples is shown by the red region. In the sample shown, the gate captured 18.5% of the events, which were taken to be the bacterial cells.

Appendix Figure S2: Optical density flattens beyond 6 h.

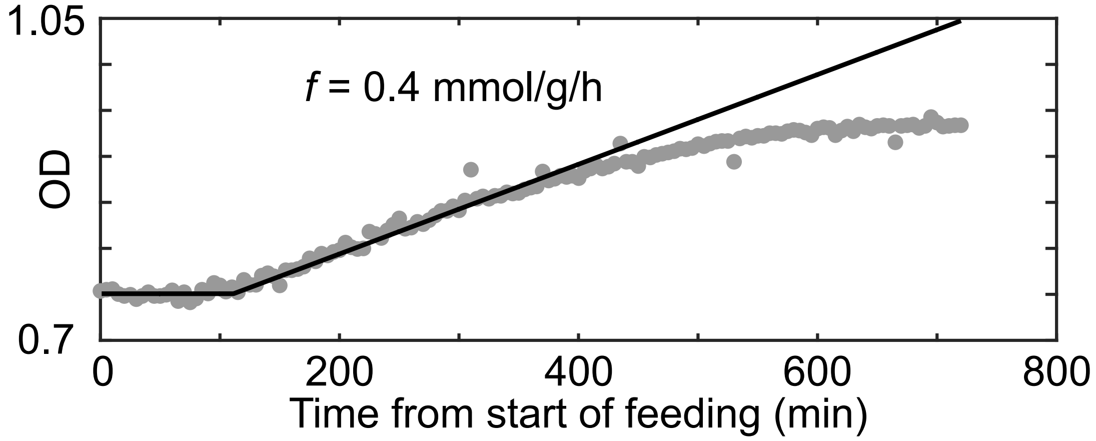

For the given glucose pulsing experiment ($f = 0.4$ mmol/g/h), optical density (OD) was measured beyond the normal 6 h. Measurements are indicated by grey dots. Beyond the default experiment time of 6 h, the OD begins to flatten and ceases linear increasing. The black line indicates the predicted OD when no flattening is assumed. The prediction was based off the empirical model of **Figure 2A** (as described in **Methods**) and the calculated division yield from **Figure 3**.

Appendix Figure S3: Additional real-time metabolomics data.

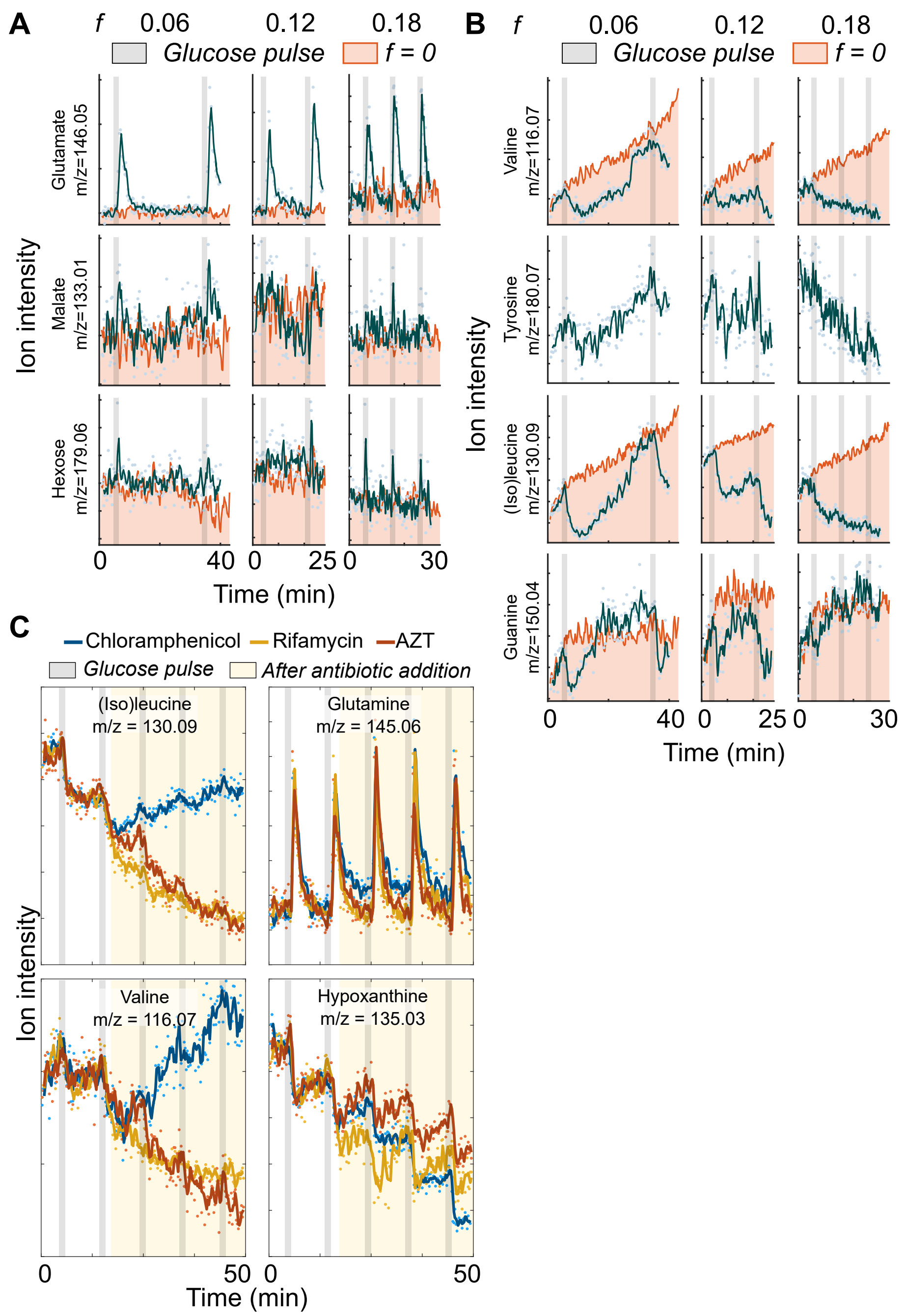

A Other central metabolites exhibited concentration spikes with glucose pulses at non-division TI feedrates (f = 0.06, 0.12, and 0.18 mmol/g/h). The TI feedrate is abbreviated as f (units: mmol glucose/g dry cell weight/hour). Glucose pulses are

indicated by the grey bars, and the pink region shows a no pulse control. Dots are ion intensity measurements. Solid lines are a moving average filter of the measured ion intensity.

B Accumulated valine, tyrosine, (iso)leucine, and guanine depleted and recovered after pulse occurrence suggesting protein and nucleic synthesis.

C Other amino acids and hypoxanthine were affected by corresponding antibiotics (f = 0.18 mmol/g/h). Antibiotics were added one minute after the second pulse (yellow region). Chloramphenicol (blue) inhibits protein biosynthesis, rifamycin (orange) inhibits RNA polymerase, and azidothymidine (AZT; red) inhibits DNA synthesis. The ion for tyrosine could not be annotated for the f = 0 mmol/g/h measurement.

Appendix Figure S4: Antibiotic metabolomics data with no antibiotic control.

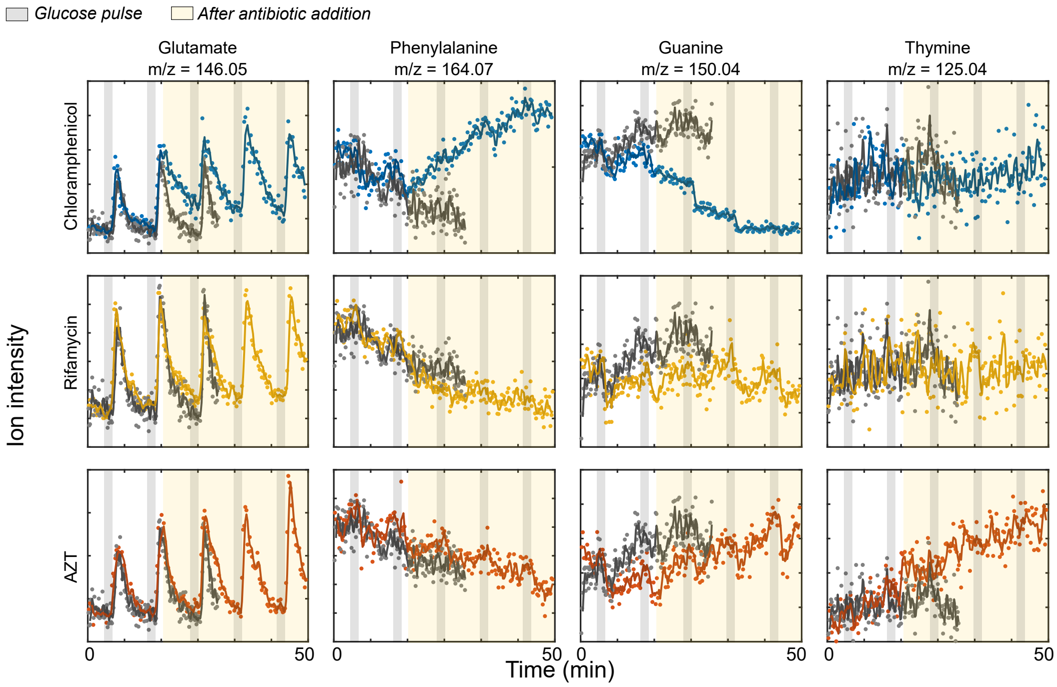

Data from **Figure 4C** is plotted against the no antibiotic control condition ($f = 0.18$ mmol/g/h from **Figure 4A**). Black dots indicate the ion intensity for the no antibiotic condition and the solid lines indicate the moving average filter of the ion intensity.

Appendix Figure S5: The crp and pdhR mutant strains have no lag.

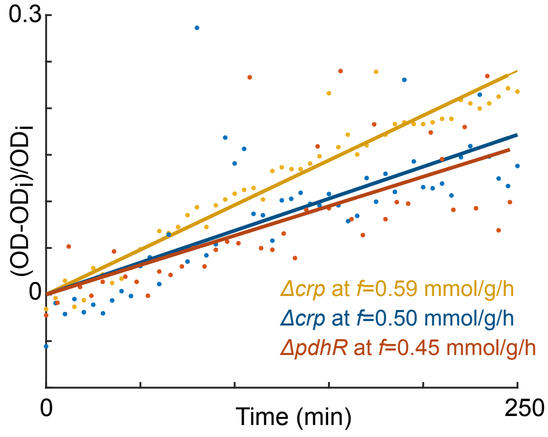

At normally lag-inducing TI feedrates (**Figure 2A**), a strain with genetic deletion of *crp* or *pdhR* showed no lag phase with glucose pulse feeding.

Appendix Figure S6: FtsZ volumetric concentration over time during the transition into starvation.

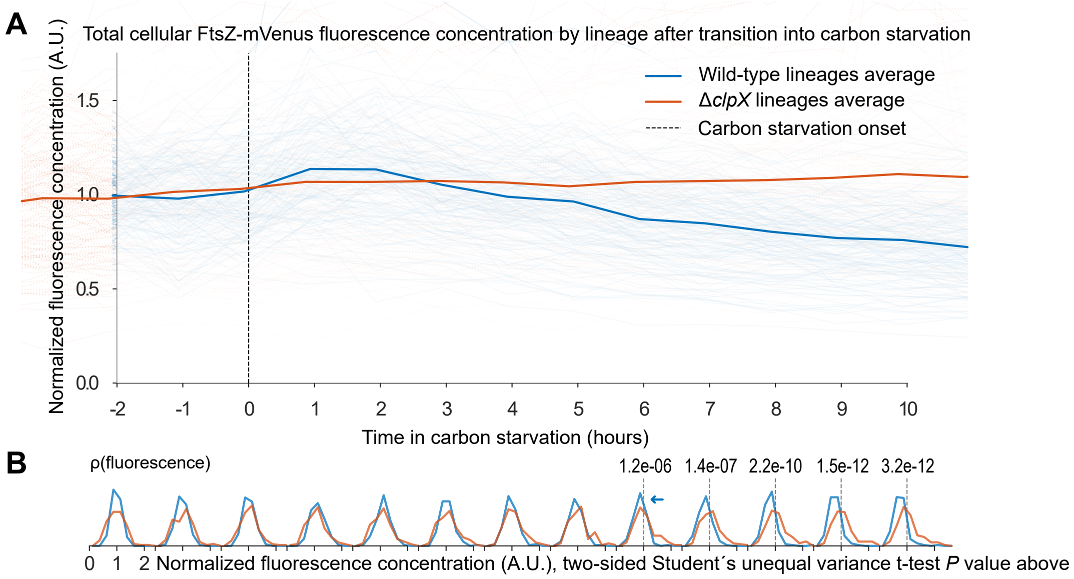

Here, the data from **Figure 8** is shown as the cellular volumetric concentration of FtsZ over time for wild-type and $\Delta clpX$ cells.

A Thin individual lines show the fluorescent concentration per cell in a single lineage (wild-type in solid blue, $n = 154$. $\Delta clpX$ in dotted red, $n = 144$). Thick solid lines are the time average across individual lineages for each subset (wild-type in blue, ΔclpX in red). Fluorescent signals are normalized to the average value for their respective subset at time 0.

B Distributions of fluorescence concentration for each subset at times corresponding to the top panel. Student's t-test P value shown for when distributions differ with significance level $\alpha = 0.01$.

Appendix Figure S7: FtsZ supplementation reduces the critical rate for division.

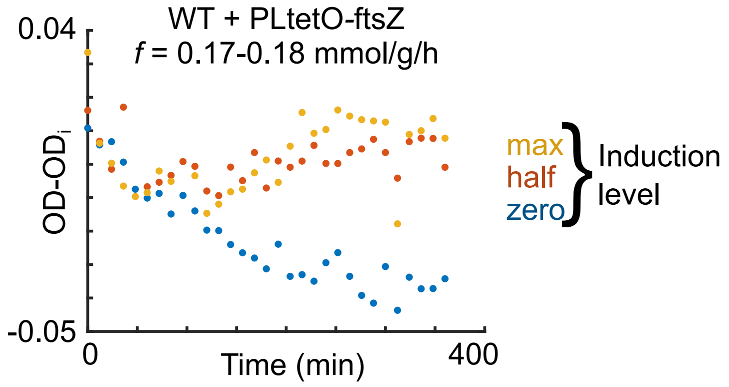

Induction of FtsZ from a plasmid allowed for division at a feedrate (f = 0.17-0.18 mmol/g/h), which is below the critical rate in the control strain (f = 0.2 mmol/g/h). For induction, max, half, and zero correspond to addition of 50, 10, and 0 ng/µL of doxycycline respectively. The TI feedrate is abbreviated as f.

Appendix Figure S8: Division is limited by FtsZ also under “natural” starvation.

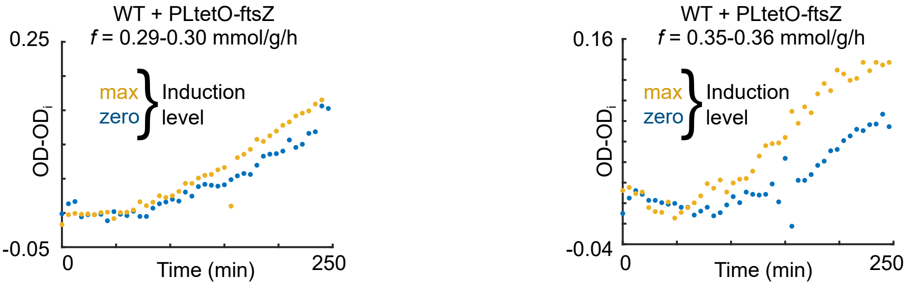

In contrast to the previous experiments with a sudden, artificially induced starvation of cells harvested from the mid exponential growth phase, here we allowed cells to enter a more natural stationary phase upon depletion of the supplied limiting glucose. After optical density stabilization, cells remained for 2 to 6 h in starvation, before adding the inducer and the initiating he glucose pulsing experiment at the indicated TI feedrates. FtsZ was titrated via plasmid-based, inducible expression. For induction, max and zero correspond to addition of 50 and 0 ng/µL of doxycycline respectively. The TI feedrate is abbreviated as *f*.

Appendix Figure S9: Titration curve of inducible plasmid with GFP.

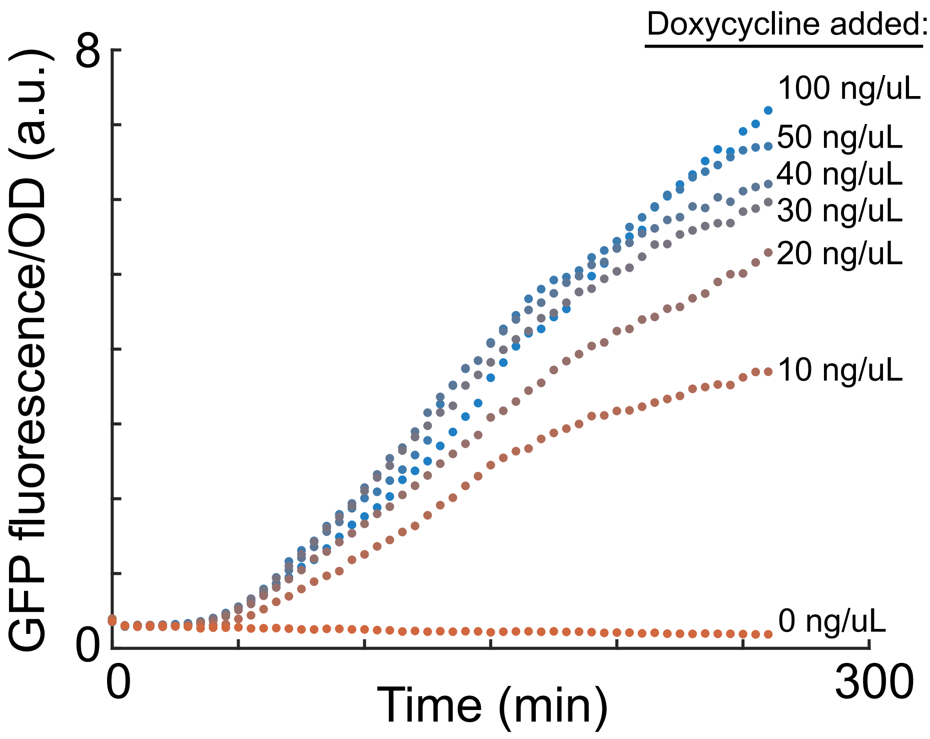

The parent plasmid pJKR-L-tetR [9] was used to determine the appropriate induction level for all titration experiments. GFP expression is driven by the titratable pLtetO promoter. Normalized GFP levels are shown for different levels of inducer doxycycline over time for microplate cultivations. Time at 0 indicates the addition of doxycycline and start of plate reader measurement. For titration experiments, 50 ng/µL was selected as high, 10 ng/µL as half, and 0 ng/µL as zero expression.

2 Supplemental Information - Development and parametrization of the FtsZ model

2.1 Introduction

This section discusses how the FtsZ model was developed and parameterized with values from previous literature. We started with the most basic model possible for the FtsZ threshold activation of division and used a differential equation to describe how FtsZ abundance changes. Starting with the equation from [12], we considered just the synthesis and degradation of FtsZ toward its changing abundance. We assumed dilution effects to be neglible because the cells are not dividing. Furthermore, we expect the number of FtsZ per cell to dictate division occurrence, not the intracellular concentration. For synthesis, we assumed that the yield of FtsZ on carbon is constant and, therefore, can be approximated as a TI feedrate dependent synthesis (αf):

$$\frac{d[\text{FtsZ}]}{dt} = \alpha f - \frac{V_{\max}[\text{FtsZ}]}{K_m + [\text{FtsZ}]} \tag{1}$$

Considering the abundance of FtsZ throughout stationary phase [10], we found that the level remains roughly constant (700 copies/cell) between 1 day and 3 days in stationary phase. This suggests that FtsZ never fully depletes during stationary phase, and that our model was oversimplified. To account for this, we posed an additional term for the basal synthesis of FtsZ (α_0):

$$\frac{d[\text{FtsZ}]}{dt} = \alpha_0 + \alpha_1 f - \frac{V_{\max}[\text{FtsZ}]}{K_m + [\text{FtsZ}]} \tag{2}$$

2.2 Closed Form Solution for the Michaelis-Menten Equation

A closed form solution to time-dependent enzyme kinetics, based on the Michaelis-Menten rate law, was first described in 1997 by Schnell and Mendoza [11]. Using the quasi-steady-state approximation, the differential equation that describes the concentration of a substrate S that is degraded by and enzyme with a maximum velocity of $V_{\max}$ is given by:

$$\frac{d[\text{S}]}{dt} = -\frac{V_{\max}[\text{S}]}{K_M + [\text{S}]} \tag{3}$$

Solving this equation for $[S](t)$ yields:

$$[\text{S}](t) = K_M \cdot W\left[\frac{[\text{S}]_0}{K_M}\exp\left(\frac{[\text{S}]_0 - V_{\max}\, t}{K_M}\right)\right] \tag{4}$$

Where $[\text{S}]_0$ is the value of [S] at time $t = 0$, and $W[\cdot]$ is the *Lambert-W* function (also known as the *omega* function or the *product logarithm*) and is defined as the inverse of the function $z \to z \cdot e^z$ (see [3]).

In our work, however, this solution is not sufficient, since we are dealing with constant positive production on top of the enzymatic degradation, i.e. $\alpha_0 + \alpha_1 f$. Therefore, we modified equation 3 to be:

$$\frac{d[\mathrm{S}]}{dt} = v_{\mathrm{in}} - \frac{V_{\max}[\mathrm{S}]}{K_M + [\mathrm{S}]} \tag{5}$$

The solution in this more general case is:

$$[\mathrm{S}](t) = [\mathrm{S}]_\infty + (K_M + [\mathrm{S}]_\infty) \cdot W\left[\frac{[\mathrm{S}]_0 - [\mathrm{S}]_\infty}{K_M + [\mathrm{S}]_\infty} \;\exp\left(\frac{[\mathrm{S}]_0 - [\mathrm{S}]_\infty - (V_{\max} - v_{\mathrm{in}})t}{K_M + [\mathrm{S}]_\infty}\right)\right] \tag{6}$$

where we define $[\mathrm{S}]_\infty \equiv K_M \frac{v_{\mathrm{in}}}{V_{\max} - v_{\mathrm{in}}}$, i.e. the value of [S] at steady-state (or $t \to \infty$).

Note that if $v_{\mathrm{in}} > V_{\max}$, the value of $[\mathrm{S}]_\infty$ will be negative, indicating that the system has no steady-state (and [S] continues to grow indefinitely). Nevertheless, equation 6 holds even in such cases, except that we need to use the lower branch of the Lambert-W function (usually denoted W_{-1}).

It is sometimes useful to consider the inverse function of $[\mathrm{S}](t)$, as we will soon see for predicting the lag time using [FtsZ] levels. Note that since the differential equation (5) is time invariant, there is a unique solution for the time difference $\Delta t = t_1 - t_0$, given the initial and final concentrations $[\mathrm{S}](t_0)$ and $[\mathrm{S}](t_1)$. The solution is given by:

$$\Delta t = \frac{1}{v_{\mathrm{in}} - V_{\max}} \cdot \left([\mathrm{S}](t_1) - [\mathrm{S}](t_0) - (K_M + [\mathrm{S}]_\infty)\ln\left(\frac{[\mathrm{S}](t_1) - [\mathrm{S}]_\infty}{[\mathrm{S}](t_0) - [\mathrm{S}]_\infty}\right)\right) \tag{7}$$

2.3 Applying the Closed Form Solution

In the case of [FtsZ], we could now apply equation 6 which will be of the following form:

$$[\mathrm{FtsZ}](t) = [\mathrm{FtsZ}]_\infty + (K_M + [\mathrm{FtsZ}]_\infty) \cdot W_i\left(a\, e^{a - b\, t}\right) . \tag{8}$$

where

$$\begin{aligned} [\mathrm{FtsZ}]_\infty &\equiv K_M \frac{\alpha_0 + \alpha_1 f}{V_{\max} - \alpha_0 - \alpha_1 f} \\ a &\equiv \frac{[\mathrm{FtsZ}]_0 - [\mathrm{FtsZ}]_\infty}{K_M + [\mathrm{FtsZ}]_\infty} \\ b &\equiv \frac{V_{\max} - \alpha_0 - \alpha_1 f}{K_M + [\mathrm{FtsZ}]_\infty} \end{aligned} \tag{9}$$

and $W_i = W_0$ in case $V_{\max} > \alpha_0 + \alpha_1 f$ and $W_i = W_{-1}$ otherwise.

In order to answer the question what is the time t at which [FtsZ] accumulate from an initial concentration (F_I) to the threshold concentration required for division (F_T), we could use the inverse formula, i.e. equation 7:

$$t_{\mathrm{lag}} = \frac{1}{\alpha_0 + \alpha_1 f - V_{\max}} \cdot \left(F_T - F_I - (K_M + [\mathrm{FtsZ}]_\infty)\ln\left(1 + \frac{F_T - F_I}{F_I - [\mathrm{FtsZ}]_\infty}\right)\right) . \tag{10}$$

As one would expect, this function diverges ($t_{\text{lag}} \to \infty$) when $[\text{FtsZ}]_\infty \to F_T$. Since $[\text{FtsZ}]_\infty$ is a function of the TI feedrate, we could solve for f from equation 2 and find that this happens when

$$f_{\text{critical}} = \frac{1}{\alpha_1}\left(V_{\max}\frac{F_T}{K_M + F_T} - \alpha_0\right).$$

For any $f < f_{\text{critical}}$, the production rate of FtsZ is too low and its level would never reach F_T.

Before using this closed form solution to predict lag times, we had to first find the values for the system parameters, namely α_0, α_1, K_M, $V_{\max}$, F_I, and F_T.

2.4 Parametrization

We first parametrized the degradation term using *in vitro* measurements from previous work [2]. We adapted data from Figure 1F within [2] to find the turnover number of FtsZ into the ClpXP protease complex. The data shows the degradation of FtsZ over 90 minutes depending on the concentration of ClpX in 25 μL total volume. We considered two points seemingly within the linear range of degradation:

ClpXP concentration [μM]	pmol FtsZ degraded/90 min	turnover [min^{-1}]
0.05	5	0.044
0.30	40	0.059

Roughly, we have a turnover number of 0.05 min^{-1}. For the actual $V_{\max}$, we need to consider the number of active ClpXP within *E. coli.* From [4], we know that ClpX is stochiometrically limiting toward formation of the ClpXP complex. Therefore, to calculate $V_{\max}$, we can simply consider the number of ClpX in *E. coli.* In starved conditions, there is approximately 200 copies of ClpX per cell [10]. Our $V_{\max}$ is approximated to be $200 \cdot 0.05 = 10$ [$\text{cell}^{-1}\text{min}^{-1}$].

Unfortunately, K_M is difficult to resolve using data from [2]. We, therefore, just used the same value from an earlier, non-specific protein degradation kinetics study [12] where $K_M = 600$ [cell^{-1}].

We could now use the final steady-state condition to calculate the basal synthesis term (α_0). Per the observation that FtsZ maintains a steady-state abundance of 700 copies per cell in stationary phase, which means that $[\text{FtsZ}]_\infty = 700$ [cell^{-1}], when $f = 0$.

$$\begin{aligned} [\text{FtsZ}]_\infty &= K_M\frac{\alpha_0 + \alpha_1 \cdot 0}{V_{\max} - \alpha_0 - \alpha_1 \cdot 0} \\ \alpha_0 &= V_{\max}\frac{[\text{FtsZ}]_\infty}{K_M + [\text{FtsZ}]_\infty} = 5.4 \ [\text{cell}^{-1}\text{min}^{-1}] \end{aligned} \tag{11}$$

We then had enough information to predict how FtsZ depletes during starvation without feeding. Per [10], we see that number of FtsZ under growing conditions is about 2000 copies/cell. We took this value to be the FtsZ concentration both at the onset of

starvation as well as the threshold F_T needed to induce cell division. Using the above parameters from literature and plugging them into equation (6), we calculated that FtsZ levels after 2 hours of starvation would deplete to 1740 copies/cell. This value was taken to be the starting FtsZ at the onset of pulsing, i.e. $F_I = 1740$ [cell^{-1}]. The values for all the model parameters are summarized in Supplementary Table S5.

2.5 Fitting α_1

Now, the only missing parameter was α_1, i.e. the yield of FtsZ from fed carbon (in units of $\left[\frac{\text{cell}^{-1}\text{min}^{-1}}{\text{mmol glc/g DCW/h}}\right]$). Since there is no available data for this relationship, we fitted the value of α_1 based on our TI feedrate versus lag time data and the results from equation (10), as illustrated in Supplementary Fig S10. Since the lag time spreads for lower feedrates, we applied a log transform on the lag times prior to fitting. We did not consider data point where no visible lag time was detected because our FtsZ model assumes $t_{\text{lag}} > 0$. We found that $\alpha_1 = 12.9 \left[\frac{\text{cell}^{-1}\text{min}^{-1}}{\text{mmol glc/g DCW/h}}\right]$.

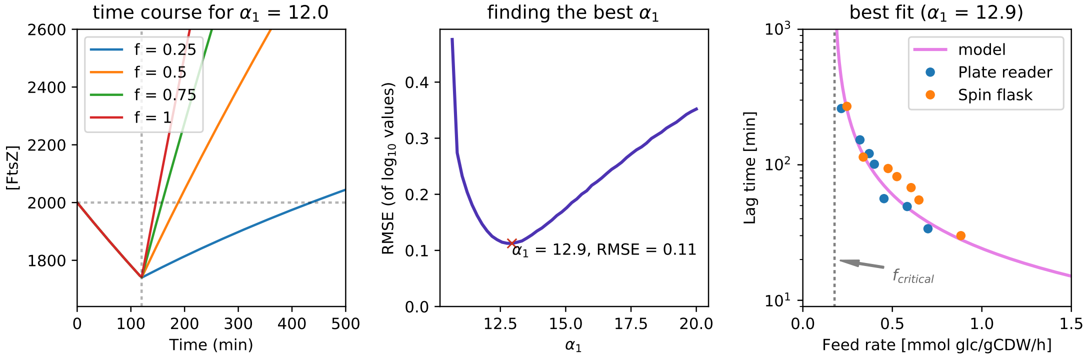

Appendix Figure S10: Fitting α_1. Given a set of parameters, our model simulates the value of [FtsZ] over time. We then fitted the value of α_1 to give the best estimate for the measured lag times (as a function of TI feedrate f). α_1 is given in units of $\left[\frac{\text{cell}^{-1}\text{min}^{-1}}{\text{mmol glc/g DCW/h}}\right]$.

We could now rewrite the lag time as a direct function of the TI feedrate f:

$$t_{\text{lag}}(f) = \frac{F_I - F_T}{V_{\text{max}} - \alpha_0 - \alpha_1 f} - \frac{K_M V_{\text{max}}}{(V_{\text{max}} - \alpha_0 - \alpha_1 f)^2} \ln\left(1 + \frac{(F_T - F_I)(V_{\text{max}} - \alpha_0 - \alpha_1 f)}{F_I(V_{\text{max}} - \alpha_0 - \alpha_1 f) - K_M(\alpha_0 + \alpha_1 f)}\right)$$

and calculate the critical TI feedrate:

$$f_{\text{critical}} = \frac{1}{\alpha_1}\left(V_{\text{max}} \frac{F_T}{K_M + F_T} - \alpha_0\right) = 0.16 \text{ mmol glc/g DCW/h}\,. \tag{12}$$

This is very close to our experimentally measured threshold of non-division (over the first 6 hours), which was at 0.2 mmol glc/g DCW/h.

Note that although $t_{\text{lag}}(f)$ seems to have a singularity point at $f = \frac{V_{\max}-\alpha_0}{\alpha_1}$ (in our case it is equal to 0.355 mmol glc/g DCW/h), the function is continuous finite at the entire range of $(f_{\text{critical}}\ ,\ \infty)$.

2.6 Taking the pulsing into account

It is important to remember that our solution for the estimated lag time (equation 10) assumes that after the starvation phase, there is a steady production of [FtsZ] $(\alpha_0 + \alpha_1 f)$. However, our experimental system provides a pulse of glucose every 2-10 minutes, and the cells consume that glucose within ≈ 0.2 minutes (assuming a maximal glucose uptake rate of 10 mmol glc/g DCW/h [7]).

In order to check whether the smooth simplification (denoted the *smooth* model) alters the predicted lag phases, we redid our calculations without this assumption, by precisely tracking the changes in FtsZ production rates during the pulse (i.e. while glucose is present) and between the pulses (i.e. while the glucose level is 0). The results are presented in Supplementary Figure S11, and show that the effect is negligible.

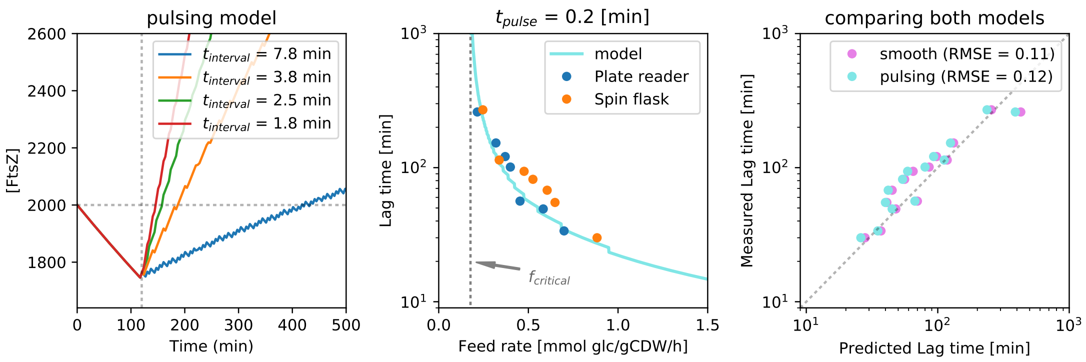

Appendix Figure S11: Analytical solution for a pulsing input function.

Finally, we compared the lag time predictions of the *smooth* and *pulsing* models, assuming the same average TI feedrate. In addition, we solved the ODE system numerically by integrating over time and finding the point where [FtsZ] crosses the threshold F_T. We conclude that the differences between all three models are negligible (Supplementary Figure S12).

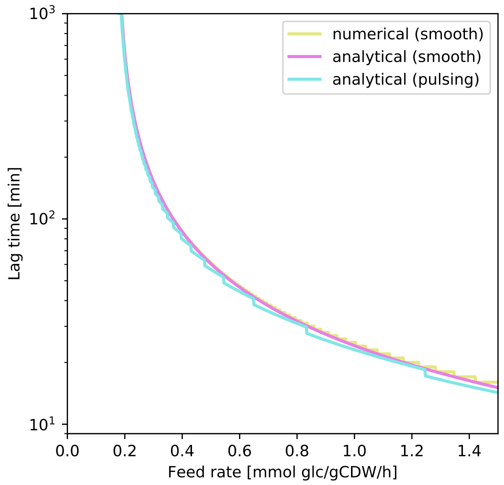

Appendix Figure S12: All three models give very similar predictions for the lag time.

All code used for parametrization and model generation is available in Supplementary Material or at `https://github.com/karsekar/pulsefeeding-analysis`.

3 Supplemental Tables

Appendix Table S1: Summary information for wild-type pulse feed experiments.

Calculated TI Feedrate (mmol/g/h)	Pulsing system	Initial OD from fit	Lag time from fit (min)	Threshold linear fit R^2	Linear growth rate after lag (1/h)	Pulse freq.$^{-1}$ (minutes)	Total glucose fed during lag (mmol/g)
0.215	Plate reader	0.70	260.16	0.607	0.021	9.98	0.93
0.246	Spin flask	0.79	270.00	0.852	0.021	7.67	1.11
0.319	Plate reader	0.78	152.81	0.974	0.030	5.99	0.81
0.337	Spin flask	1.11	113.97	0.882	0.025	4.00	0.64
0.371	Plate reader	0.74	121.00	0.965	0.039	5.49	0.75
0.400	Plate reader	0.75	101.02	0.976	0.038	4.99	0.67
0.453	Plate reader	0.78	56.28	0.985	0.039	4.24	0.43
0.476	Spin flask	0.74	93.89	0.955	0.030	4.25	0.75
0.525	Spin flask	0.68	81.85	0.938	0.032	4.17	0.72
0.583	Plate reader	0.74	49.22	0.987	0.059	3.49	0.48
0.605	Spin flask	0.83	67.86	0.987	0.044	3.00	0.68
0.648	Spin flask	0.92	55.00	0.996	0.045	2.50	0.59
0.699	Plate reader	0.78	33.75	0.983	0.066	2.75	0.39
0.782	Spin flask	0.70	0.00	0.953	0.044	2.75	0.00
0.883	Spin flask	0.85	30.00	0.970	0.048	2.00	0.44
1.026	Plate reader	0.63	0.00	0.961	0.092	2.31	0.00
1.097	Spin flask	0.82	0.00	0.914	0.041	1.67	0.00
1.237	Spin flask	0.69	0.00	0.962	0.059	1.75	0.00

For the units, mmol is mmol of glucose, and g is grams dry cell weight of *E. coli*.

Appendix Table S2: C^{13} Labeled fraction of measured amino acids and glycogen in washed, hydrolyzed extract.

Compound [# of native carbons in fragment]	*Fraction of [+X] fragment (n = 3, biological) at given TI feedrate* 0 mmol/g/h		0.06 mmol/g/h		0.12 mmol/g/h		0.18 mmol/g/h	
Class 1[+] - Non-accumulating amino acids								
Alanine [3]	0.002	± 0.002	0.025	± 0.003	0.058	± 0.007	0.060	± 0.003
Aspartate [4]	0	± 0	0.014	± 0.001	0.038	± 0.001	0.060	± 0.003
Glutamate [5]	0.0006	± 0.0001	0.017	± 0.001	0.039	± 0.001	0.065	± 0.002
Glycine [2]	0.0013	± 0.0007	0.022	± 0.001	0.04	± 0.01	0.070	± 0.003
Proline [4]	0.0005	± 0.0003	0.0061	± 0.0002	0.013	± 0.001	0.018	± 0.001
Serine [2]	0.001	± 0.001	0.026	± 0.002	0.039	± 0.008	0.057	± 0.002
Threonine [4]	0	± 0	0.008	± 0.001	0.022	± 0.002	0.039	± 0.004
Class 2[+] - Accumulating amino acids								
Isoleucine [5]	0.003	± 0.001	0.003	± 0.001	0.006	± 0.003	0.00013	± 0.00001
Leucine [5]	*[+5] not detected*							
Lysine [5]	*[+5] not detected*							
Methionine [4]	*[+4] not detected*							
Phenylalanine [8]	*[+8] not detected*							
Tyrosine [9]	*[+9] not detected*							
Valine [4]	0.002	± 0.001	0.0010	± 0.0002	0.002	± 0.001	0.008	± 0.002
Glycogen [5]	0.0004	± 0.0001	0.00028	± 0.00002	0.0007	± 0.0003		

All measurements are after 6 hours of pulsing. Values are the mean ± standard error of independent biological replicates ($n = 3$ for amino acid samples, $n = 2$ for glycogen). +Class designation is from [6].

Appendix Table S3: Strains used in this study

Strain	Genotype	Description
BW 25113	Δ(araD-araB)567 Δ(rhaD-rhaB)568 ΔlacZ4787 (::rrnB-3) hsdR514 rph-1	Parent strain from Keio Collection [1]. Used as wild-type (WT).
Δ*crp*	Same as BW 25113 with Δcrp. Kanamycin marker was excised from corresponding strain from [1].	*crp* deletion strain.
Δ*pdhR*	Same as BW 25113 with ΔpdhR. Kanamycin marker was excised from corresponding strain from [1].	*pdhR* deletion strain.
Δ*clpX*	Same as BW 25113 with ΔclpX. Strain from Keio collection [1]. Kan marker was not removed.	*clpX* deletion strain.
Δ*clpP*	Same as BW 25113 with ΔclpP. Strain from Keio collection [1]. Kan marker was not removed.	*clpP* deletion strain.
SJ1725	ΔftsZ::[ftsZ55-mVenus-56] F- lambda- ilvG- rfb-50 rph-1 MG1655	Strain with *ftsZ* replaced with ftsZ-mVenus fusion. Derived from [8]
SJ1741	ΔftsZ::[ftsZ55-mVenus-56] ΔclpX::[FRT kan FRT] F- lambda- ilvG- rfb-50 rph-1 MG1655	Same as SJ1725 with additional *clpX* deletion.

Appendix Table S4: Plasmids used in this study

Plasmid	Description	Reference
epd-icd	Constitutive GFP plasmid with SC101 backbone.	[5]
pJKR-L-tetO	Parent plasmid for titrated synthesis of GFP with tetracycline-based inducer. SC101 backbone.	[9]
pPLtetO-PdhR	Derived from pJKR-L-tetO. Titrates synthesis of PdhR with tetracycline-based inducer.	This study.
pPLtetO-FtsZ	Derived from pJKR-L-tetO. Titrates synthesis of FtsZ with tetracycline-based inducer.	This study.
pPLtetO-ClpX	Derived from pJKR-L-tetO. Titrates synthesis of ClpX with tetracycline-based inducer.	This study.
pPLtetO-FtsA	Derived from pJKR-L-tetO. Titrates synthesis of FtsA with tetracycline-based inducer.	This study.
pPLtetO-FtsB	Derived from pJKR-L-tetO. Titrates synthesis of FtsB with tetracycline-based inducer.	This study.
pPLtetO-FtsN	Derived from pJKR-L-tetO. Titrates synthesis of FtsN with tetracycline-based inducer.	This study.
pPLtetO-FtsL	Derived from pJKR-L-tetO. Titrates synthesis of FtsL with tetracycline-based inducer.	This study.

Appendix Table S5: System parameters

Parameter	Value	Units
K_M	600	cell^{-1}
$V_{\max}$	10	$\text{cell}^{-1}\text{min}^{-1}$
α_0	5.4	$\text{cell}^{-1}\text{min}^{-1}$
α_1	12.9	$\frac{\text{cell}^{-1}\text{min}^{-1}}{\text{mmol glc/g DCW/h}}$
F_I	1740	cell^{-1}
F_T	2000	cell^{-1}
f_{critical}	0.16	mmol glc/g DCW/h

References

[1] T. Baba, T. Ara, M. Hasegawa, Y. Takai, Y. Okumura, M. Baba, K. A. Datsenko, M. Tomita, B. L. Wanner, and H. Mori. Construction of Escherichia coli K-12 in-frame, single-gene knockout mutants: the Keio collection. *Mol. Syst. Biol.*, 2:2006.0008, 2006.

[2] J. L. Camberg, J. R. Hoskins, and S. Wickner. ClpXP protease degrades the cytoskeletal protein, FtsZ, and modulates FtsZ polymer dynamics. *Proc. Natl. Acad. Sci. U.S.A.*, 106(26):10614–10619, Jun 2009.

[3] R M Corless, G H Gonnet, D E G Hare, D J Jeffrey, and D E Knuth. On the LambertW function. *Adv. Comput. Math.*, 5(1):329–359, 1 December 1996.

[4] C. M. Farrell, A. D. Grossman, and R. T. Sauer. Cytoplasmic degradation of ssrA-tagged proteins. *Mol. Microbiol.*, 57(6):1750–1761, Sep 2005.

[5] L. Gerosa, K. Kochanowski, M. Heinemann, and U. Sauer. Dissecting specific and global transcriptional regulation of bacterial gene expression. *Mol. Syst. Biol.*, 9:658, Apr 2013.

[6] H. Link, T. Fuhrer, L. Gerosa, N. Zamboni, and U. Sauer. Real-time metabolome profiling of the metabolic switch between starvation and growth. *Nat. Methods*, 12(11):1091–1097, Nov 2015.

[7] J. M. Monk, A. Koza, M. A. Campodonico, D. Machado, J. M. Seoane, B. O. Palsson, M. J. Herrgard, and A. M. Feist. Multi-omics Quantification of Species Variation of Escherichia coli Links Molecular Features with Strain Phenotypes. *Cell Syst*, 3(3):238–251, Sep 2016.

[8] D. A. Moore, Z. N. Whatley, C. P. Joshi, M. Osawa, and H. P. Erickson. Probing for Binding Regions of the FtsZ Protein Surface through Site-Directed Insertions: Discovery of Fully Functional FtsZ-Fluorescent Proteins. *J. Bacteriol.*, 199(1), 01 2017.

[9] J. K. Rogers, C. D. Guzman, N. D. Taylor, S. Raman, K. Anderson, and G. M. Church. Synthetic biosensors for precise gene control and real-time monitoring of metabolites. *Nucleic Acids Res.*, 43(15):7648–7660, Sep 2015.

[10] A. Schmidt, K. Kochanowski, S. Vedelaar, E. Ahrne, B. Volkmer, L. Callipo, K. Knoops, M. Bauer, R. Aebersold, and M. Heinemann. The quantitative and condition-dependent Escherichia coli proteome. *Nat. Biotechnol.*, 34(1):104–110, Jan 2016.

[11] S Schnell and C Mendoza. Closed form solution for time-dependent enzyme kinetics. *J. Theor. Biol.*, 187(2):207–212, 21 July 1997.

[12] K. Sekar, A. M. Gentile, J. W. Bostick, and K. E. Tyo. N-Terminal-Based Targeted, Inducible Protein Degradation in Escherichia coli. *PLoS ONE*, 11(2):e0149746, 2016.
