# Supplementary material for: Synthesis and degradation of FtsZ quantitatively predict the first cell division in starved bacteria
Source: Mol Syst Biol. 2018 Nov 5;14(11):e8623. doi: 10.15252/msb.20188623 (PMC6217170; doi:10.15252/msb.20188623)
Supplement: Supplementary file 2 — Expanded View Figures PDF [file MSB-14-e8623-s002.pdf]

Expanded View Figures

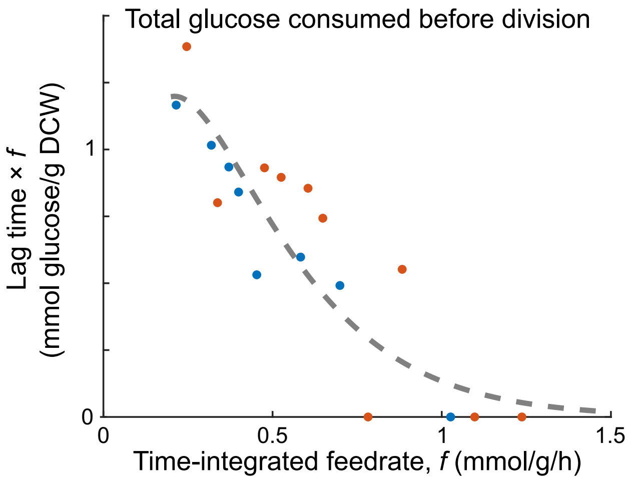

Figure EV1. Total glucose fed during lag does not determine division occurrence.

Data from Fig 2A were replotted to total amount of glucose fed before division (lag duration times the TI feedrate) versus the TI feedrate. The total amount of glucose needed to trigger division is not constant and increases for decreasing TI feedrate.

© 2018 The Authors

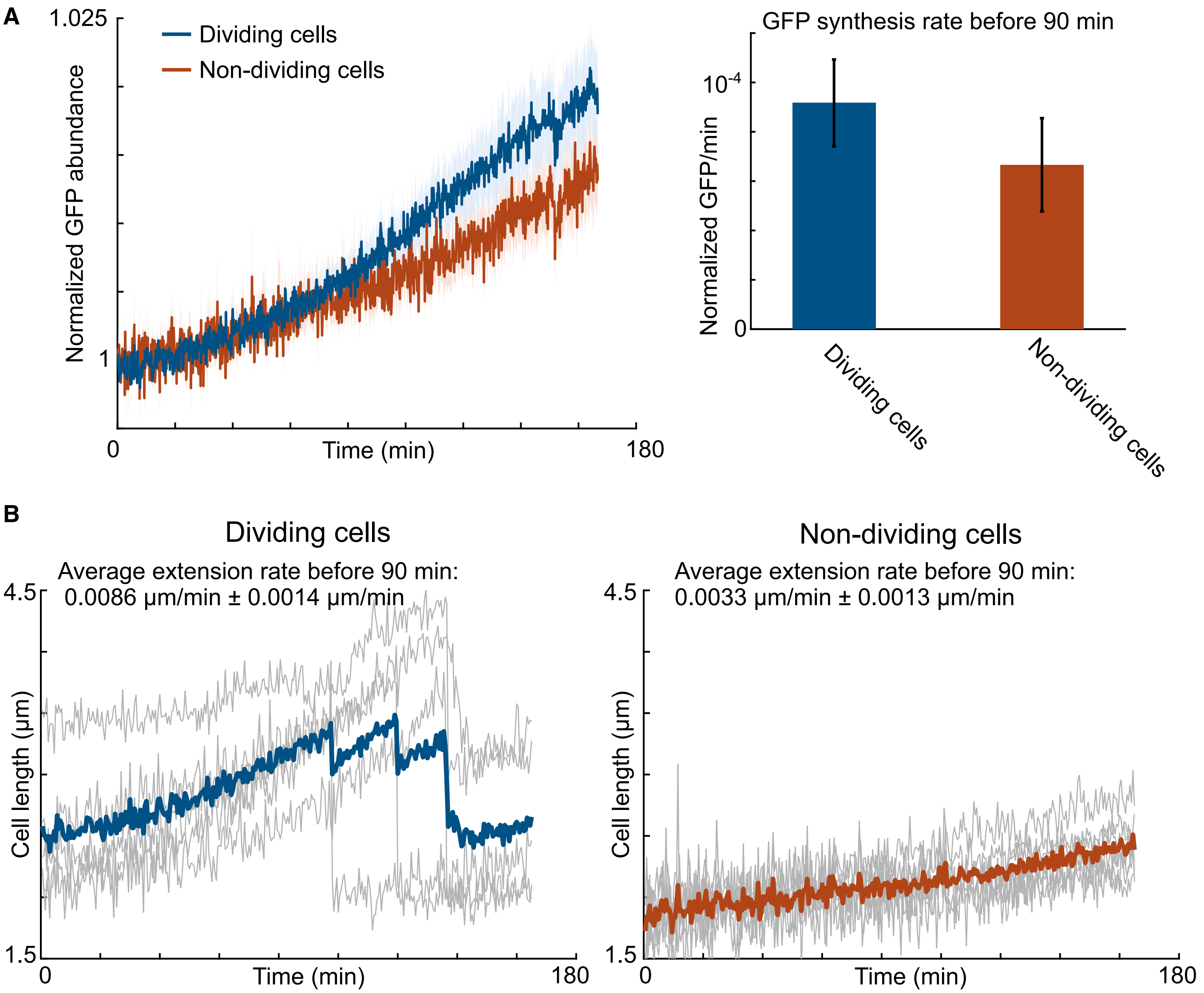

Figure EV2. All cells make protein and increase in size with glucose feed.
Cell length and GFP expression were measured using the microfluidic-based microscopy platform for glucose pulsing periods of 4 min. Two groups were determined: a dividing group (blue), where cells divided within the given experiment time (5 h), and a non-dividing group (red), where the cells did not divide within the 5 h.

A Both dividing and non-dividing cells made protein as indicated by signal from constitutive GFP expression. Solid lines indicate the moving average for each given group. Lightly shaded regions indicate average ± standard error of the cells (n = 5). GFP synthesis rate was calculated before division (~90 min) by using linear fitting. The bar graph shows the GFP synthesis rate for the both dividing and non-dividing cells. Values are mean ± standard error of independent cells (n = 5).

B Cell length increased with pulsing in both the dividing versus non-dividing subpopulation. Solid lines (blue and red) indicate the average of the individual cells. Gray lines indicate lengths of individual cells over time. Average cell extension rate before division was calculated using linear fitting for each cell. Values are mean ± standard error of independent cells (n = 5).

 © 2018 The Authors

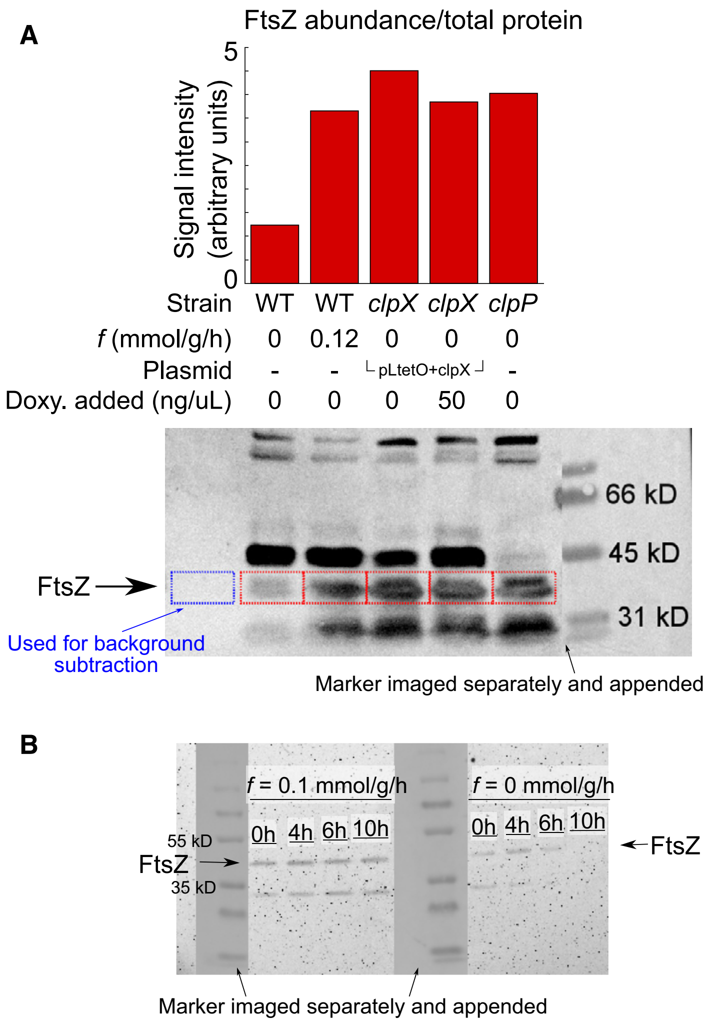

Figure EV3. Western blots validate ClpXP-mediated degradation of FtsZ *in vivo* during starvation and synthesis of FtsZ with glucose pulsing.

1.5 ng total protein was loaded into each lane. Protein marker was imaged separately with bright-field and appended to blot with exact positioning.

A After 16 h, relative FtsZ abundance (from blot directly below) is much lower in wild-type cells without any glucose pulsing (f = 0 mmol/g/h) compared to conditions with glucose pulsing (f = 0.12 mmol/g/h) or in strains absent of ClpXP machinery (*clpX* and *clpP*). Supplemental synthesis of ClpX within a *clpX* strain via expression off the pLtetO + clpX plasmid shows less FtsZ when ClpX synthesis is on (50 ng/µl doxycycline added) versus off (0 ng/µl doxycycline). Bordered areas were quantified with MATLAB 2015b. The subtracted background is indicated by the blue border.

B A time course immunoblot shows depletion of FtsZ in the no pulse condition (f = 0 mmol/g/h) versus the pulsing condition (f = 0.1 mmol/g/h) across 10 h. Time indicates sampling points from the beginning of pulsing (2 h into glucose starvation) for both experiments.

© 2018 The Authors

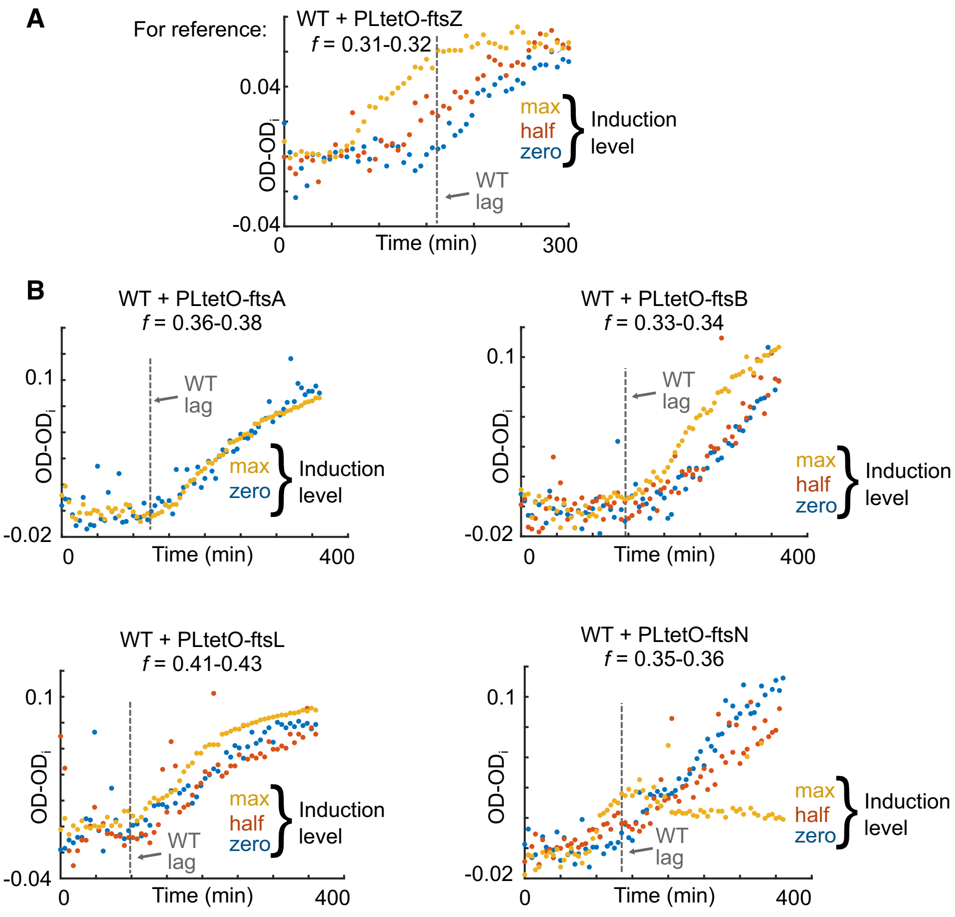

Figure EV4. Titrations of other division proteins support FtsZ as the division limitation.

A Figure 9B is reproduced here as reference and shows the decrease of lag time monotonic to the FtsZ induction level. All proteins were titrated via plasmid-based, inducible expression. For induction, max, half, and zero correspond to addition of 50, 10, and 0 ng/μl of doxycycline, respectively. Units of TI feedrate *f* are mmol/g/h.

B Lag times do not decrease with induction level of other division proteins (FtsL, FtsB, and FtsA). FtsB and FtsL induction minimally increased more division after lag end. Lag time decreases with FtsN induction, and total division is decreased as shown by the lower final OD.

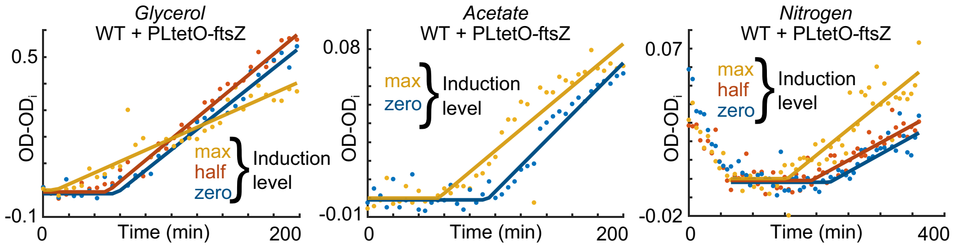

Figure EV5. FtsZ-limited division applies for various nutrient limitations.

Supplemental FtsZ titration reduced the lag time in starved cells that were pulse-fed with the limiting nutrients glycerol, acetate, or ammonium. For induction, max, half, and zero correspond to addition of 50, 10, and 0 ng/μl of doxycycline, respectively. For the glycerol experiment, cells were grown in glycerol to exponential phase prior to starvation. Pulse concentration was 38 μM glycerol, and the TI feedrate was 0.6 mmol glycerol/g DCW/h where OD 1 corresponds to 0.54 g DCW/L (Gerosa *et al*, 2015). For the acetate experiment, cells were grown on acetate as the sole carbon source prior to starvation and pulse feeding. Pulse concentration was 88 μM sodium acetate at a TI feedrate of 3.0 mmol acetate/g DCW/h where OD 1 corresponds to 0.44 g DCW/L (Gerosa *et al*, 2015). For the ammonium experiment, cells were grown in M9 glucose media, then starved in media without ammonium, and consequently pulse-fed. Pulse concentrations were 1.5 μM ammonium sulfate, and the TI feedrate was 0.045 mmol ammonium sulfate/g DCW/h.

 © 2018 The Authors
