# Supplementary figures and images for: Synthesis and degradation of FtsZ quantitatively predict the first cell division in starved bacteria
Source: Mol Syst Biol. 2018 Nov 5;14(11):e8623. doi: 10.15252/msb.20188623 (PMC6217170; doi:10.15252/msb.20188623)

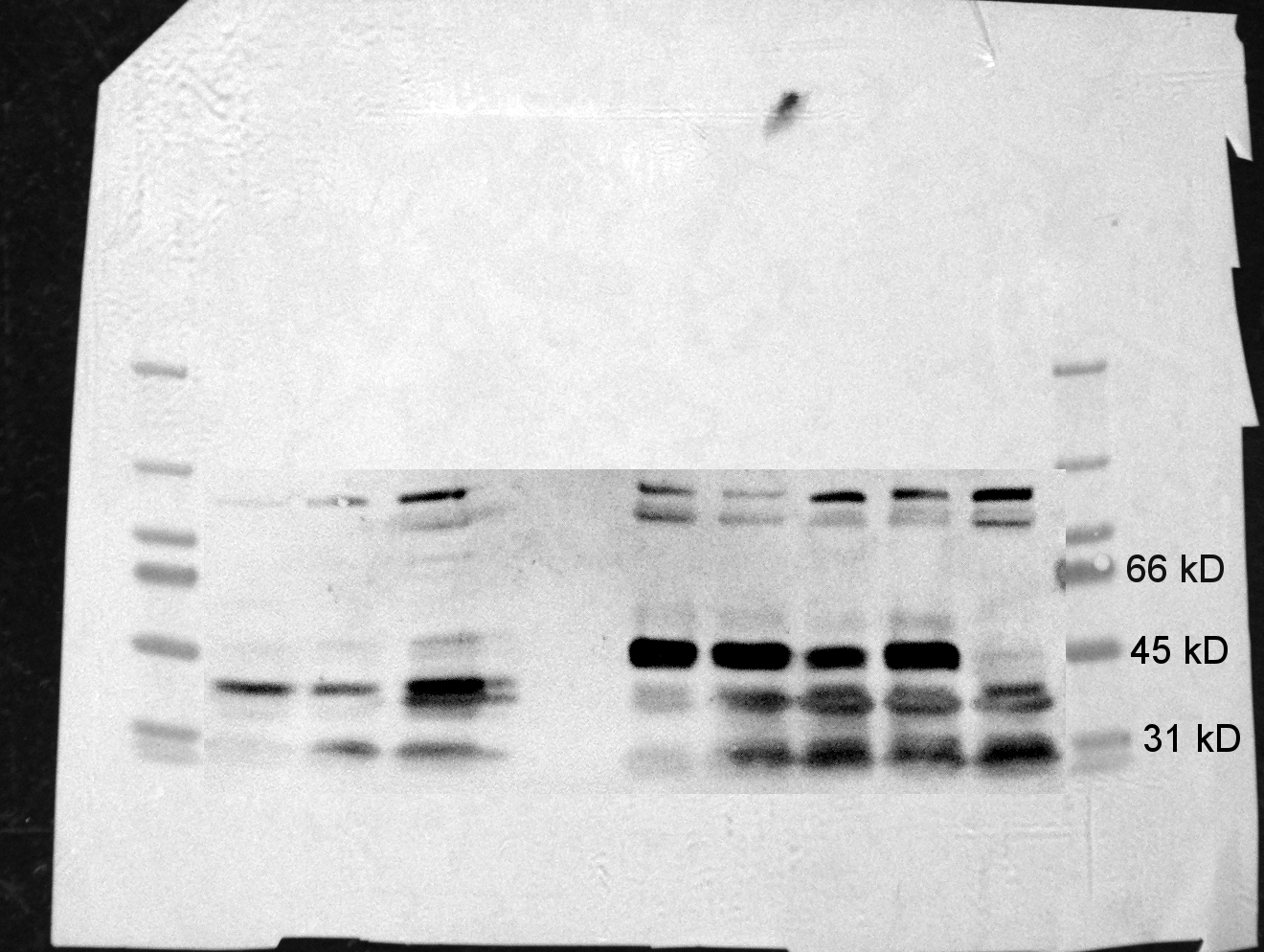

Supplement: Supplementary file 5 — Dataset EV3 [file MSB-14-e8623-s005.zip › Dataset_EV_3_pulsefeeding-analysis/12immunoblotting/blot1.bmp]
